# Effect of electronic doping and traps on carrier dynamics in tin halide perovskites†

**DOI:** 10.1039/d2mh00008c

**Published:** 2022-04-01

**Authors:** Antonella Treglia, Francesco Ambrosio, Samuele Martani, Giulia Folpini, Alex J. Barker, Munirah D. Albaqami, Filippo De Angelis, Isabella Poli, Annamaria Petrozza

**Affiliations:** Center for Nano Science and Technology @PoliMi, Istituto Italiano di Tecnologia via G. Pascoli 70/3 20133 Milano Italy annamaria.petrozza@iit.it isabella.poli@iit.it; Physics Department, Politecnico di Milano Piazza L. da Vinci, 32 20133 Milano Italy; Computational Laboratory for Hybrid/Organic Photovoltaics (CLHYO), Istituto CNR di Scienze e Tecnologie Chimiche “Giulio Natta” (CNR-SCITEC) Perugia Italy; Department of Chemistry and Biology “A. Zambelli”, University of Salerno, 84084 Fisciano Salerno Italy; Chemistry Department, College of Science, King Saud University Riyadh 11451 Saudi Arabia; Department of Chemistry, Biology and Biotechnology, University of Perugia Perugia Italy

## Abstract

Tin halide perovskites have recently emerged as promising materials for low band gap solar cells. Much effort has been invested on controlling the limiting factors responsible for poor device efficiencies, namely self-p-doping and tin oxidation. Both phenomena are related to the presence of defects; however, full understanding of their implications in the optoelectronic properties of the material is still missing. We provide a comprehensive picture of the competing radiative and non-radiative recombination processes in tin-based perovskite thin films to establish the interplay between doping and trapping by combining photoluminescence measurements with trapped-carrier dynamic simulations and first-principles calculations. We show that pristine Sn perovskites, *i.e.* sample processed with commercially available SnI_2_ used as received, exhibit extremely high radiative efficiency due to electronic doping which boosts the radiative band-to-band recombination. Contrarily, thin films where Sn^4+^ species are intentionally introduced show drastically reduced radiative lifetime and efficiency due to a dominance of Auger recombination at all excitation densities when the material is highly doped. The introduction of SnF_2_ reduces the doping and passivates Sn^4+^ trap states but conversely introduces additional non-radiative decay channels in the bulk that fundamentally limit the radiative efficiency. Overall, we provide a qualitative model that takes into account different types of traps present in tin-perovskite thin films and show how doping and defects can affect the optoelectronic properties.

## Abstract 2

Interplay of radiative and non-radiative recombination processes in tin halide perovskites as a result of doping, oxidation and defects.

## Graphical abstract

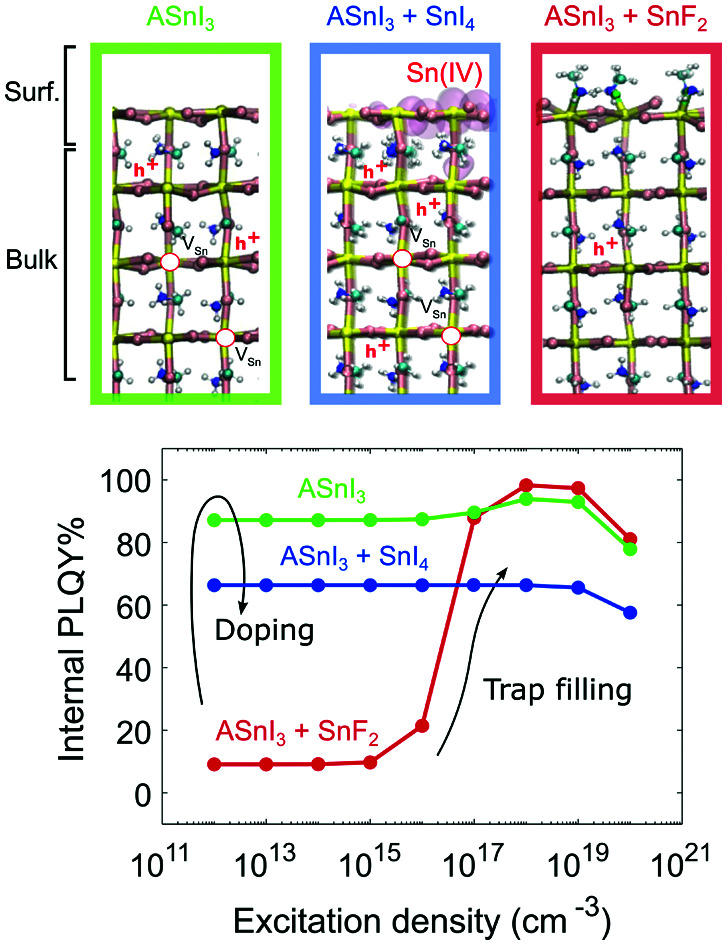

**New concepts**

We address the interplay of radiative and non-radiative processes occurring in tin-halide perovskites as a result of electronic and chemical doping, oxidation and density of trap states. A complete picture of the different processes counteracting and their effects on the optoelectronic properties of the material is still missing and is provided in this study, where carrier dynamics simulations are adopted as a useful tool to understand experimental results and identify the dominant processes occurring in different regimes.

## Introduction

Tin halide perovskites (THP) have received increasing interest as alternative candidates to lead halide perovskites (LHP) not only because of their reduced environmental toxicity,^1^ but also due to their narrower bandgap of ∼1.3–1.4 eV,^2,3^ which is very close to the ideal bandgap for single-junction solar cells.^4^ THPs have seen great improvements in terms of power conversion efficiency (PCE) and reproducibility since their launch in 2014,^5^ achieving a current record PCE of 14.7%.^6^ However, considering that the theoretical maximum efficiency with such a bandgap should exceed 30%, the ambitious target in terms of device performance is a long way from being fulfilled. One big challenge is that THPs are generally p-doped semiconductors with limited carrier diffusion lengths and short lifetimes, while the foremost studied LHPs are intrinsic.^7^ Such p-doping is believed to be caused by a self-doping process induced by highly stable shallow Sn vacancy defects,^8–11^ and by the chemical instability of the Sn element in the 2+ oxidation state and its tendency to oxidize to the 4+ state.^12–14^

Different strategies to control the intrinsic doping have been studied, such as the use of reducing agents,^15–19^ alternative solvents, ^20–22^ the addition of low dimensional features,^23–26^ passivating additives^27,28^ and the addition of extra Sn^2+^ compensation.^29–32^ Up to now, tin fluoride (SnF_2_) is the most commonly used additive to improve the film quality^33–37^ and most importantly to avoid the formation of Sn vacancies, leading to reduced background hole densities, during the film formation.^30–32^

To successfully integrate THP in reliable PV technologies, a deep understanding of the fundamental optoelectronic properties of the material in terms of defect chemistry is necessary. Although various works tried to elucidate the key optoelectronic properties of Sn-containing perovskites,^37–42^ it is important to further experimentally investigate these materials to establish the interplay between electronic doping and carrier trapping. We have recently reported that THP thin films have high external photoluminescence quantum yields (PLQY),^43^ which indicates that THPs are virtually free of active deep traps, or, in other words, that the radiative decay is more efficient than carrier trapping dynamics.

Here, we provide a comprehensive picture of the radiative and non-radiative recombination processes in FA_0.85_Cs_0.15_SnI_3_ (FACsSnI_3_) thin films. In particular, we combine recombination dynamic simulations with photoluminescence measurements and density functional theory (DFT) calculations of defects in THPs. We show that pristine FACsSnI_3_ thin films, *i.e.* those prepared without exposing the film to ambient conditions during growth, exhibit high photoluminescence quantum yields of about 20% due to the self-p-doping characteristic of the material. Contrarily, the presence of 2% Sn^4+^ impurities within the precursor solution has a critical effect on optoelectronic properties, drastically reducing the radiative efficiency and carrier lifetime. We demonstrate that Auger recombination plays a fundamental role in limiting the performances even at low photoexcitation density when the material is highly doped. The optoelectronic properties of FACsSnI_3_ thin films containing Sn^4+^ species can be recovered upon addition of small fractions of SnF_2_, due to a reduction of doping and F-passivation of Sn^4+^ defects, predicted especially at the surface. This result is supported by DFT calculations demonstrating the role of fluoride in passivating surface Sn defects. Meanwhile, Sn-rich sources should be limited due to the introduction of additional non-radiative decay channels that lead to an overall detrimental effect on optoelectronic performances in films with excess of SnF_2_. We propose a kinetic model to simulate the evolution of free and trapped carriers in doped materials as a tool for the interpretation of spectroscopic experiments of tin perovskite thin films with variable doping and trap density.

## Results and discussion

The recombination mechanisms and dynamics in FACsSnI_3_ perovskites can be explored by using a simple kinetic model that simulates the generation and recombination of charged carriers in the presence of both p-doping and deep electron traps. The full model is reported in Section S1.1 of the ESI.† It can be simplified since hole traps can be neglected (*cf.* Section S1, ESI†), therefore obtaining the following system of rate equations (eqn (1)–(3)) and initial conditions (eqn (4) and (5)) to describe the recombination dynamics of charged carriers:1
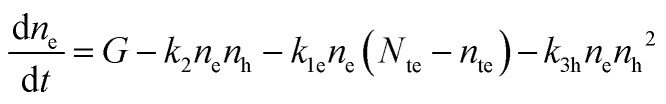
2
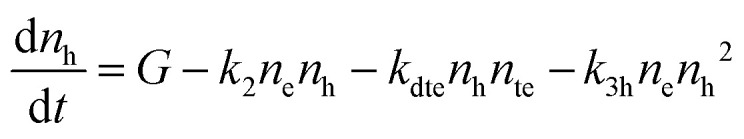
3
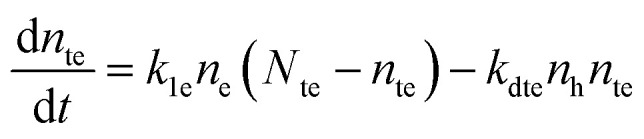

4 *n*_e_ = *n*

5 *n*_h_ = *n* + *p*_0_

where *n*_e_ and *n*_h_ are the density of electrons and holes, respectively, *n*_te_ is the density of trapped electrons, *k*_2_ is the radiative recombination rate constant, *k*_1e_ is the electron trapping rate constant, *N*_te_ is the density of available electron traps, *k*_dte_ is the rate of recombination of a trapped electron with a free hole, *G* is the photogeneration rate and *n* is the excitation density. The concentration of dopant holes *p*_0_ is included as initial condition and is considered as indistinguishable from photogenerated holes once the material is photoexcited. Finally, *k*_3h_ is the Auger recombination rate constant for hole–hole–electron events.

The system of rate equations was solved to simulate the temporal evolution of the photoluminescence signal (Fig. S1, ESI†) and to extract two important figures of merit to evaluate the optoelectronic quality of halide perovskites: the carrier lifetime (*τ*) and the internal photoluminescence quantum yield (PLQY) (*cf.* Section S1.2, ESI†).

Given the high number of free parameters in the model, it is impractical to use accurate values to simulate the expected outcomes, *i.e.*, lifetime and radiative efficiency. Therefore, starting from values reported in the literature^37,60,61^ and listed in Tables S1 and S2 in the ESI,† we study the effect of the variation of each parameter (Fig. S2, ESI†) and the effect they have if introduced in a system of gradually increasing complexity (Fig. S3–S5, ESI†). We must also emphasize that the simplified model used herein does not include all processes and non-radiative paths that might occur in real systems (photon recycling, outcoupling efficiency, *etc.*). A more detailed and thorough study should be separately done to quantify these effects.^62–64^

**Fig. 1:**
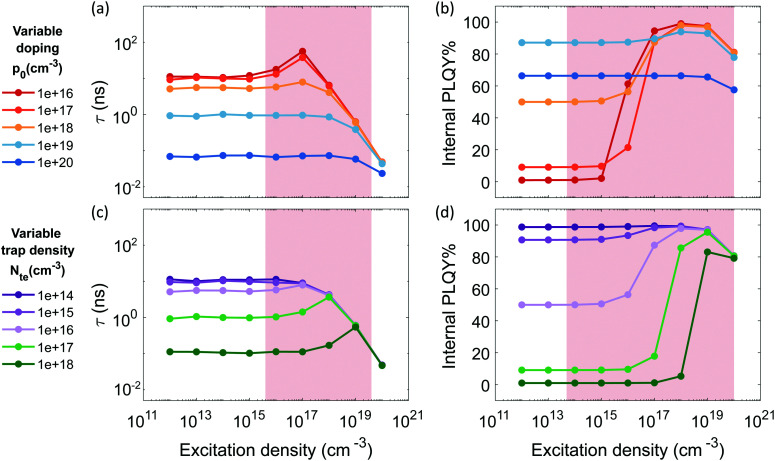
Simulated carrier lifetime (a and c) and internal PLQY (b and d) as solutions to the system of equations eqn (1)–(5). (a and b) With variable doping density and trap density fixed at 1016 cm^−3^ (parameters listed in Table S1 (ESI†)); (c and d) with variable trap density and doping density fixed at 1018 cm^−3^ (parameters listed in Table S2 (ESI†)). Shaded regions indicate the experimental measurement ranges showed in Fig. 2 and 3.

Fig. 1 reports the simulated lifetime and PLQY for the selected parameters as function of doping and trap density. Fig. 1a and b show a variable doping scenario with the carrier lifetime and PLQY simulated as a function of excitation density for a p-doped semiconductor with *p*_0_ ranging between 10^16^ and 10^20^ cm^−3^ (see Fig. S1 (ESI†) for the simulated Time-Resolved Photoluminescence TRPL decays). Table S1 (ESI†) lists the free parameters used. Here we assume a trap states density *N*_te_ of 10^16^, which is within the simulated photo-excitation range, and relevant for the PV regime. We immediately notice that the lifetime considerably drops with the doping density (Fig. 1a) due to radiative band-to-band recombination of photoexcited electrons with dopant holes (Fig. S3, ESI†). For doping densities *p*_0_ ≤ 10^18^ cm^−3^ the lifetime, which is initially flat, starts to increase when *n* becomes comparable with the density of traps *N*_te_. This increase is followed by a drop as the radiative bimolecular band-to-band recombination becomes dominant (Fig. S3, ESI†). The PLQY instead is initially flat with a baseline value that increases with doping density. Then, it increases with excitation density indicating that traps are getting filled and showing the typical trend observed in the presence of dominant deep carrier trap states (Fig. S4, ESI†). Finally it decreases for *n* ≥ 10^19^ cm^−3^ due to Auger recombination (Fig. S5 top, ESI†). Conversely, in case of medium and high doping density (*p*_0_ > 10^18^ cm^−3^) the carrier recombination dynamics fall into two regimes. For *n* < *p*_0_, monomolecular radiative recombination of electrons with dopant holes dominates, with an *n*-independent radiative lifetime; while for *n* > *p*_0_ the bimolecular recombination of photoexcited carriers dominates with a *n*-dependent decreasing lifetime. For 10^18^ cm^−3^ ≤ *p*_0_ ≤ 10^19^ cm^−3^, the carrier lifetime drops, while the PLQY keeps increasing as *p*_0_ grows. This is the result of an enhancement of the free holes background with respect to the density of carrier trapping states which boosts the radiative recombination. With *p*_0_ > 10^19^ cm^3^ Auger recombination processes become dominant, causing a decrease of both the lifetime and the PLQY at all excitation densities (Fig. S2e and S5 bottom, ESI†). Overall the high doping density completely masks all trap-induced dynamics occurring in the material.

Fig. 1c and d illustrate a variable trap density scenario with the simulated carrier lifetime and PLQY as a function of excitation density for a p-doped semiconductor (*p*_0_ = 10^18^ cm^−3^) with density of traps ranging between 10^14^ and 10^18^ cm^−3^. The parameters used for the simulation are listed in Table S2 (ESI†). We notice that increasing the density of traps causes a drastic reduction of both the lifetime and the PLQY due to a dominant non-radiative recombination over the radiative one induced by doping.

To sum up, we show that the dominant recombination path for electrons in p-doped FACsSnI_3_ materials might be either with traps or with dopant holes *via* monomolecular processes, depending on their relative value and the range of excitation density considered. If trapping dominates monomolecular trap-assisted recombination is the limiting process, inducing *n*-dependent *τ* and low PLQY values at low injection levels. If doping and trap density are comparable an interplay of the two processes is observable: while *τ* is *n*-independent, the PLQY shows high values at low injection levels with an increase as traps gets filled with increasing excitation density. When doping dominates both *τ* and PLQY result to be *n*-independent with Auger non-radiative recombination becoming the dominant process. This effect of Auger reduction of PLQY even at low excitation density (typical of device operating condition) must be carefully considered when optimizing material performances.

The developed model is a useful tool to interpret experimental data when doping and defects simultaneously affect the optoelectronic properties of the material. Therefore, we explore the recombination mechanisms and dynamics of FACsSnI_3_ perovskites by conducting steady state and time-resolved spectroscopic measurements across a range of excitation densities to unravel links between hole doping density, non-radiative recombination pathways and transport properties of THP. We first investigate the optoelectronic properties of THP by comparing pristine FACsSnI_3_ and Sn^4+^-rich FACsSnI_3_. Then, we study the interplay between doping and trapping in FACsSnI_3_ perovskites by tuning the presence of electron traps and background holes upon addition of different quantities of SnF_2_.

Previous works have shown that the intrinsic doping character of Sn-based perovskites, activated by lattice instabilities and defects, largely affects the optoelectronic properties of the material.^17,37,39,42^ In presence of O_2_, during or after the fabrication of Sn-containing perovskites, even if in low traces, Sn^2+^ gets easily oxidized and 2 holes are released into the lattice, contributing to increasing the doping (extrinsic doping).^13^ Herein, we want to investigate the effect that controlled oxidation have on the optoelectronic properties of the material. We compare FACsSnI_3_ films processed with commercially available SnI_2_ used as received and films processed by adding 2 mol% Sn^4+^ in the form of SnI_4_ (UV-VIS spectra of precursor solution are shown in Fig. S6c, ESI†), hereafter referred to as ‘pristine’ and ‘Sn^4+^-rich’, respectively, and discuss their key optoelectronic properties of absorption, carrier generation and recombination.

Both pristine and Sn^4+^-rich thin films exhibit good coverage and similar morphology, as evidenced by the scanning electron microscopy (SEM) images shown in Fig. S7a and b (ESI†), suggesting that the presence of Sn^4+^ does neither affect the growth of grains nor evidently segregate as a separate phase on the surface. X-ray diffraction (XRD) patterns (Fig. S7c, ESI†) show that both samples are highly crystalline. All peaks can be associated with a pseudocubic crystal structure, as previously reported.^38,65^ Neither broadening of diffraction peaks nor the presence of impurity phases are observed, suggesting that Sn^4+^ does not induce any clear lattice distortion.

**Fig. 2:**
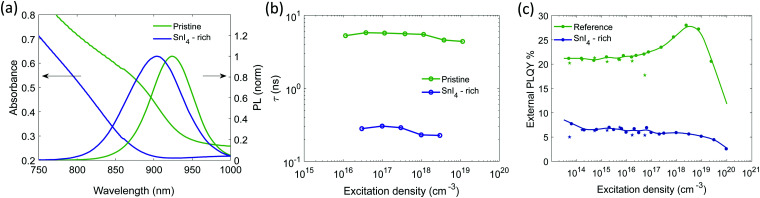
Pristine and SnI_4_-rich samples: (a) absorption spectrum and PL emission (excitation with 450 nm CW laser at 100 mW cm^−2^), (b) lifetimes extracted from single exponential fitting of the TRPL decays as function of excitation density. The fluence dependence for the SnI_4_-rich sample is measured with Transient absorbption (TA) spectroscopy, (c) external PLQY taken with increasing excitation density, experimental points indicated by a star, at excitation densities lower than 10^17^ cm^−3^ were measured with CW excitation. Experimental points indicated by dots, were measured with pulsed excitation (repetition rate 500 kHz) to avoid sample degradation. The relative value is normalized on the absolute PLQY at 1 sun (about 5 × 10^14^ cm^−3^) measured with an integrating sphere. Non normalized data points are reported in Fig. S16b (ESI†).

Fig. 2a shows the optical properties of both pristine and Sn^4+^-rich thin films. The absorption onset of the Sn^4+^-rich film is blue shifted and has a milder decrease in intensity with respect to the pristine material, which may indicate disorder induced by the presence of defects^66^ and/or heavier doping (Burstein–Moss effect), as previously reported by Herz and coworkers.^41,67^ The normalized steady-state PL emission is blue-shifted by about 70 meV and broadened compared with the pristine material, indicating larger spread of energy states at the band edges.

**Fig. 3:**
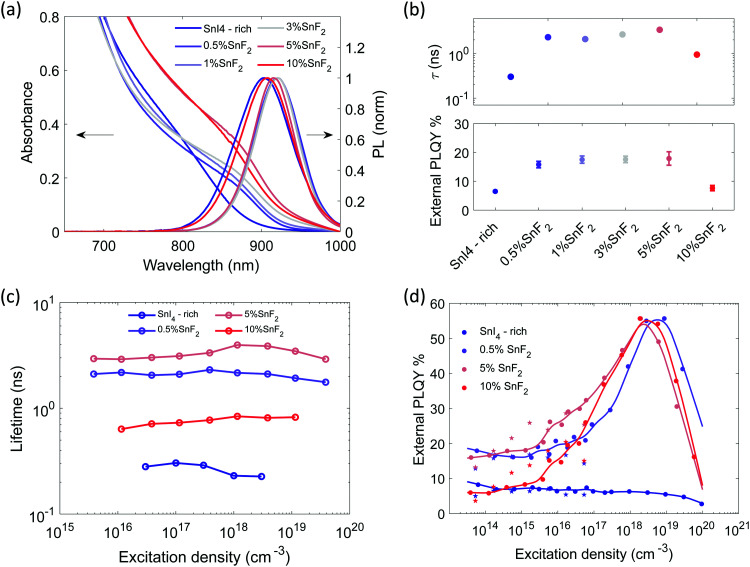
Sn^4+^-rich and with gradual increase of SnF_2_ content: (a) absorption and PL emission spectra (excitation with 450 nm CW laser at 100 mW cm^−2^), (b) top: lifetimes extracted from single exponential fitting of the TRPL decays with excitation density at about 5 × 10^17^ cm^−3^ in Fig. S15a (ESI†), bottom: external PLQY measured using an integrating sphere system, CW excitation at 373 nm and a power density of 100 mW cm^−2^, (c) lifetimes extracted from single exponential fitting of the TRPL decays as function of excitation density. The fluence dependence for the SnI_4_-rich sample is measured with TA, (d) external PLQY taken with increasing excitation density. Experimental points indicated by a star, at excitation densities lower than 10^17^ cm^−3^ were measured with CW excitations. Experimental points indicated by dots were measured with pulsed excitation (repetition rate 500 kHz) to avoid sample degradation. The relative value is normalized on the absolute PLQY at 1 sun (about 5 × 10^14^ cm^−3^) measured through an integrating sphere. Non normalized data points are reported in Fig. S16b (ESI†).

Fig. 2b and c show the lifetime extracted from TRPL decay and external PLQY of both pristine and Sn^4+^-rich films. At excitation densities of 100 mW cm^−2^, the pristine sample shows a high PLQY, exceeding 20% (Fig. S8a for statistics, ESI†) and a lifetime of about 5 ns with a dynamic that can be fitted by a single exponential (Fig. S9a, ESI†). The lifetime is *n*-independent at low excitation densities and starts to decrease at *n* > 10^18^ cm^−3^. The PLQY is also *n*-independent for *n* < 10^17^ cm^−3^, then, at higher excitation densities, it starts growing up to a plateau followed by a sharp drop. This behavior suggests a scenario where the sample has a level of doping < 10^17^ cm^−3^, the excitation density value at which trap mediated phenomena are not masked anymore by the free background holes. This means that at low excitation densities the PL dynamics are driven by a monomolecular decay which boosts the radiative recombination and masks the carrier traps given the relatively high density of background free holes. Then, at higher excitation densities, a trap filling process shows up which is quickly damped by Auger recombination. The Sn^4+^-rich sample shows a drastically reduced PLQY of only 7% (Fig. S8b, ESI†) and a lifetime of around 100 ps (estimated with transient absorption measurement and shown in Fig. S9a and b, ESI†). Lifetime and PLQY result to be *n*-independent in the examined excitation density range. According to the simulations in Fig. 1a and b, this observation is an indication of increased doping density where Auger recombination involving dopant holes masks any trap mediated process by flattening and decreasing the PLQY at all excitation densities. This experimental observation is supported by a recent computational report showing that Sn^4+^ is not stable in the bulk, spontaneously releasing two holes to the valence band and increasing the p-doping of the material.^13^ At the same time it is not possible to exclude a simultaneous increment in trap density as it would contribute similarly in the decrease of lifetime and PLQY, as shown in Fig. 1c and d. Furthermore, the conductivity of the film only slightly increases upon addition of Sn^4+^ species (Fig. S10, ESI†), which supports the increase in doping and introduction of defect states that affects the effective carrier mobility.

To study the interplay between doping and trapping in FACsSnI_3_ perovskites, we need to monitor the PLQY and the lifetime across a range of excitation densities systematically tuning both the doping level and the trap states density. This can be done by adding different quantities of SnF_2_. We start from Sn^4+^-rich precursor solutions and we add 0.5, 1, 3, 5 and 10% SnF_2_ (Fig. S11, ESI†), in the following referred as ‘Sn^4+^-rich’, ‘0.5% SnF_2_’, ‘1% SnF_2_’, ‘3% SnF_2_’, ‘5% SnF_2_’, ‘10% SnF_2_’ respectively.

SEM images of the films without and with SnF_2_ are reported in Fig. S12a–c (ESI†). Full coverage is preserved and slight enlargement of grain size is observed upon addition of SnF_2_. Films with SnF_2_ present brighter streaks on the surface of the grains, which might be an indication of preferential growth in the direction perpendicular to the substrate, as recently reported for mixed Sn/Pb compositions.^68^ XRD patterns (Fig. S12d and S13, ESI†) show that the presence of SnF_2_ does not introduce impurity peaks preserving the pseudocubic crystal structure. Fig. 3a shows the absorption and PL spectra of THP films with different SnF_2_ contents. With the introduction of SnF_2_, we observe a redshift of the absorption onset (Fig. S14a, ESI†). The PL peak of films containing up to 5% SnF_2_ are narrower (Fig. S14b, ESI†) and red shifted by 20 meV (Fig. S14a, ESI†) with respect to the Sn^4+^-rich sample, indicating a decrease in energetic disorder, possibly through the inhibition of Sn vacancy formation and therefore a reduction of background doping.^38^ Indeed, the conductivity of SnF_2_-containing films is found to be lower with respect to pristine FACsSnI_3_ materials (Fig. S10, ESI†). Further increasing SnF_2_ content to 10% broadens the PL, suggesting that excess of SnF_2_ can increase the energetic disorder, overcompensating the beneficial effects deriving from the reduction in background hole density.^38^

The presence of only 0.5% of SnF_2_ increases the lifetime by about one order of magnitude and considerably improves the PLQY, which rises from 7 to 18% at excitation density comparable to 1 sun, as shown in Fig. 3b. At higher excitation density (Fig. 3d) a typical trap filling process is revealed. The concomitant enhancement of lifetime and PLQY indicates a reduction of doping and carrier trap states based on the simulations in Fig. 1, indeed the system is not anymore in the regime of Auger-induced decrease of PLQY at all excitation densities. The density of electronic doping and traps is further reduced when increasing the SnF_2_ up to 5%. This is evident from the longer measured lifetime (Fig. 3c) and in the anticipation at lower excitation densities of the growth of the PLQY *via* trap filling towards the band to band recombination regime (Fig. 3d). It is interesting to notice that in presence of SnF_2_ the doping level becomes less relevant with respect to charge trapping dynamics, therefore, as the excitation density increases traps get filled and 50% of PLQY is reached. As the excitation density increases Auger kicks in damping the enhancement of PLQY. Upon further increase to 10%, the lifetime is reduced and the PLQY drops to 8% (Fig. S14c for statistics, ESI†), which indicates an increase in non-radiative recombination channels. To substantiate our results we apply the model to experimental lifetime and PLQY of the pristine, SnI_4_ -rich, 0.5% SnF_2_, 5% SnF_2_ and 10% SnF_2_ (Fig. S17, ESI†). The initial values are reported in Tables S1 and S2 (ESI†), while the output parameters are included in Table S3 (ESI†). The trend of the doping density and defect density upon addition of SnI_4_ and SnF_2_ is in line with the discussion above.

In the previous section, we have seen that adding SnF_2_ to a Sn^4+^-rich film drastically improves its PLQY. In particular, an amount as small as 0.5% is able, already, to reduce carrier trap density. To better understand the role of Sn^4+^ and the effect of SnF_2_ we discuss the thermodynamics of surface defects in THPs, since it has been demonstrated that Sn^4+^ is not stable in the bulk of tin halide perovskites.^69^ To this end, we employ of state-of-the-art DFT calculations (*cf.* Computational Details).

**Fig. 4:**
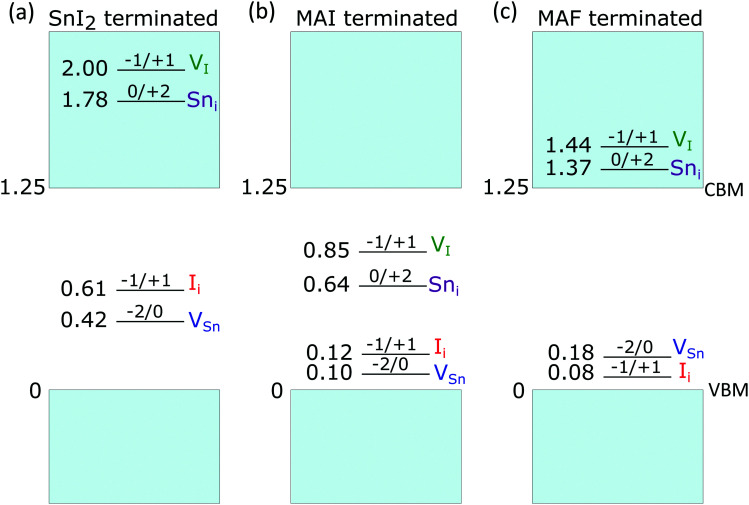
Lowest thermodynamic charge transition levels of point defects on the SnI_2_, MAI and MAF(001) terminated MASnI_3_ surfaces. All energies are referred to the valence band maximum (VBM) of the respective surface model. The notation *q*/*q*′ used in the figure refers to charge transition levels (*q*/*q*′) as defined in Computational details.

Fig. S19 (ESI†) shows the defect formation energy diagram of defects in SnI_2_-terminated MASnI_3_ surface. As observed for MAPbI_3_,^47^ the surface terminated with metal iodide (Fig. 4a) is prone to suffer from hole trapping defects such as V_Sn_ and I_i_, while electron traps are deactivated as their charge transition levels are found to lie above the conduction band of the material. It has been shown that a surface tin vacancy defect can enhance hole localization on undercoordinated tin atoms, if compared to the pristine surface, thus triggering the formation of Sn^4+^ through a disproportionation reaction.^13^ Sn^4+^ defects, on an unpassivated surface, act as electron traps increasing non-radiative recombination. However, the stabilization of hole localization on the SnI_2_-terminated surface may also lead to surface iodide oxidation, previously observed for MAPbI_3_ and MAPb_0.5_Sn_0.5_I_3_,^14,47^ with the formation of the tri-iodide moiety I_3_^−^, for both V^0^_Sn_ and I_i_^+^, upon capture of two holes. In fact, for V^0^_Sn_, we here calculate formation energies for this species to be only 0.1 eV higher than those pertaining Sn^4+^ (Fig. S20, ESI†). However, we also find that, upon injection of a single hole, this is preferentially localized on an undercoordinated surface tin atom, upon contraction of five Sn–I bonds. This moiety (Fig. S20, ESI†) represents a singly-oxidized precursor to surface Sn^4+^, and is found to be 0.28 eV more stable than the oxidized iodide, *i.e.* a I_2_^−^ moiety (so-called V-center),^70^ which instead precedes the formation of I_3_^−^ (Fig. S20, ESI†). In contrast, for I_i_^+^, iodide oxidation is favoured with I_3_^−^ being 0.41 eV more stable than a surface Sn^4+^. Overall, we can point out a competitive behaviour in the oxidation of either surface iodide or tin, in analogy with that previously observed for mixed lead/tin perovskites.^14^

We then investigate a fully passivated surface, modeled by considering a full MAI coverage, for which a progressive destabilization of hole traps is envisaged (Fig. S19, ESI†). Hole traps, such as V_Sn_ and I_i_ feature only shallow energy levels (Fig. 4), mainly as a consequence of a sizable up-shift of the valence band edge (Table S4, ESI†). We further verify that these defects are indeed unable to trap holes on this surface termination (Fig. S21, ESI†). In contrast, electron traps are found to be stabilized, thus essentially preserving the defect physics of the bulk material (Fig. 4b and Fig. S19, ESI†).^8^ In particular, Sn_i_ and V_I_ can localize up two electrons, *via* the formation of a Sn–Sn bond^69^ (Fig. S22 (ESI†) for examples of structural configurations). Charge localization entails an energy gain that can be quantified by defining a binding energy *E*_b_ = *E*^*q*^[*X*]_deloc_ − *E*^*q*^[*X*]_loc_ as the total-energy difference between the defect with delocalized charges and that featuring charge localization, due to the dimer formation. From Table S5 (ESI†), we note that localization of two electrons is energetically favoured for both Sn_i_ and V_I_, in line with previous observations for the bulk material.

Next, we investigate the possible effect of fluoride on the electronic properties of the surface by substituting surface I with F, thus effectively achieving a MAF termination. In fact, it is known that fluoride ions from SnF_2_ tend to localize preferentially at the surface,^71^ since the large mismatch between Sn–I and Sn–F bonds partially hinders their assimilation in the bulk. When simulating the substitution reaction *n*MAI_surf_ + *n*/2SnF_2_ → *n*MAF_surf_ + *n*/2SnI_2_, we find it to be energetically favoured with each I/F substitution entailing an energy gain of ∼0.60 eV. This effect is due to the high electronegativity of F resulting in a strong interaction with sub-surface Sn atoms and surface MA cations. We then calculate the formation energies of the point defects for the MAF-terminated slab and we find that both hole and electron traps are inactivated, with charge transition levels either close to the valence band edge of the material or above the conduction band edge (Fig. 4c). These observations cannot be ascribed to band shift. In fact, the terminal MAF layer upshifts the band edges by ∼0.6 eV with respect to the MAI termination but this does not translate into an increased stability range of electron traps. Instead we observe that, while the formation energies of I defects are almost unchanged if compared to both the bulk material and the MAI-terminated slab (Fig. S19b, ESI†), those of Sn defects are drastically increased. We can therefore attribute to F the beneficial role in passivating surface Sn defects. In particular, Sn vacancies for tin atoms bonded to surface F are destabilized in virtue of the stronger Sn–F bond. Instead, surface Sn interstitials are unfavoured by the larger steric hindrance provided by the MAF layer (Fig. S22 for selected structural models, ESI†). By analysing the atomistic structures of the defects, we observe that, when the Sn–Sn bond is formed, its length is noticeably longer than that recorded on the MAI terminated surface (Table S5, ESI†). This leads to minimal values of binding energies or even to a complete destabilization of the localized system (*i.e.* negative values of *E*_b_).

Overall, by combining computational results with the experiment, it is possible to rationalize the interplay between bulk and surface effects in determining the photo-physics of Sn^4+^-rich and %SnF_2_ tin iodide perovskites. Calculations show how surface V_Sn_ and I_i_, associated with the occurrence of oxidized Sn^4+^ and I_3_^−^ species, respectively, can act as deep hole traps on unpassivated SnI_2_-rich surfaces, which can be favoured when a fraction of SnI_4_ is included in the precursors solution. These middle-gap defects, which instead are only shallow in the pristine bulk material and on passivated surfaces, can be related to the enhanced trap density evidenced by spectroscopy measurements. Upon addition of a fraction of SnF_2_, self-p doping is mitigated by reduction of tin vacancies but at the same time also surface hole and electron traps are passivated, thus justifying the simultaneous reduction of both doping and trap densities. However, growing the tin perovskite in Sn-rich conditions lowers the formation energy of tin interstitials and iodine vacancies, both capable of trapping electrons in the bulk material.^69^ This is in agreement with the enhanced efficiency of non-radiative decay channels observed at high percentage of SnF_2_ addition.

## Conclusions

We have identified radiative and non-radiative processes occurring in tin halide perovskites as a function of doping, oxidation and density of trap states. We propose a qualitative kinetic model to simulate the evolution of free and trapped carriers in doped materials as a tool for the interpretation of spectroscopic experiments of tin perovskite thin films with variable doping and trap density. Pristine materials exhibit high radiative efficiency due to an intrinsic doping level that is higher or comparable to the density of carrier trap states. In contrast, when the density of Sn^4+^ species becomes relevant, higher energetic disorder, reduced lifetime and PLQY are observed. This suggests a dominance of Auger-like processes even at low excitation densities that masks the potential increase in trap density. Supported by computational studies, we propose a simultaneous increase of doping and trap density. The introduction of SnF_2_ increases the lifetime and radiative efficiency by reducing the background doping and passivating electronic trap states. Computational studies suggest the beneficial role of F in passivating Sn defects, supporting the observed increased PLQY upon addition of SnF_2_. However, this is true only for low concentration of the additive. In fact we experimentally demonstrate that an excess of SnF_2_ creates a Sn-rich growth condition that results in the dominant effect of non-radiative decay channels over the beneficial role of F in passivating the thin film surface, with an overall detrimental effect on optoelectronic performances.

## Experimental

### Materials and methods

*N*,*N*-Dimethylformamide (DMF, anhydrous, 99.8%), dimethyl sulfoxide (DMSO, anhydrous, ≥99.9%) anisole (anhydrous, 99.7%) were purchased from Sigma-Aldrich; tin(ii) iodide (SnI_2_, for Perovskite precursor) was purchased from Tokyo Chemical Industry (TCI); tin(ii) fluoride (SnF_2_, 97.5%) and tin(iv) iodide (SnI_4_, 99.998%) were purchased by Alfa Aesar. All chemicals were used without any further purification. Glass substrates were cleaned in acetone and isopropyl alcohol (IPA) for 10 min by sonication. The cleaned glass substrates were treated with oxygen plasma for 10 min before any further deposition. Thin-film perovskite deposition was done in a N_2_-filled glovebox and thin-films were glass encapsulated immediately after thermal annealing (in the glovebox to avoid oxygen).

#### Sn-Based thin films

To make Sn-based (FA_0.85_Cs_0.15_SnI_3_) thin-film perovskite the precursor solution (concentration of 1.2 M) was prepared in mixed solvents of DMF and DMSO with a volume ratio of 4 : 1. The molar ratios for FAI/CsI was 0.85 : 0.15 and the molar ratio of (FAI + CsI)/SnI_2_ was 1 : 1. Different concentration of SnF_2_ (0.5, 1, 3, 5 and 10 mol% relative to SnI_2_) was added in the precursor solution. The precursor solution was stirred at 60 °C for 30 min and then filtered through 0.20 μm PTFE membrane before use. The perovskite films were deposited with one-step spin-coating procedures at 4000 rpm for 50 s. Anisole (100 μl) was dropped on the spinning substrate at 25 s before the end of the procedure. The substrates were annealed at 120 °C for 20 min.

### Characterization

XRD patterns were recorded with a Bruker D8 Advance diffractometer with Bragg–Brentano geometry equipped with a Cu Kα1 (*λ* = 1.544060 Å) anode, operating at 40 kV and 40 mA. All the diffraction patterns were collected at room temperature, with a step size of 0.05 in symmetric scan reflection mode and an acquisition time of 1 s. XRD patterns were recorder on thin films in an inert environment by means of a Bruker airtight specimen holder with dome like X-ray transparent cap, for environmentally sensitive materials.

SEM images were obtained using a MIRA3 TESCAN microscope with an accelerating voltage of 4 kV. Perovskite films were prepared on ITO substrates.

UV-vis steady state absorption spectra were measured on perovskite thin films deposited on bare glass using a UV/VIS/NIR spectrophotometer Lambda 1050, PerkinElmer, in the wavelength range 350–1100 nm, a step size of 1 nm.

Steady State PL (+Rel PLQY) was performed with a 450 nm c.w. diode laser (Oxxius, unfocused beam diameter of 1 mm) as the excitation source for CW characterization. An amplified femtosecond laser (Light Conversion Pharos) generated pulses of ∼280 fs centered at 515 nm with 500 kHz repetition rate was used as the excitation source for pulsed excitation. Photoluminescence was collected in reflection mode at a right angle from the excitation line and focused into a fiber coupled to a spectrometer (Ocean Optics Maya Pro 2000) with an intensity of ∼100 mW cm^−2^ for absolute characterization. PL was measured in air on glass encapsulated samples. For relative PLQY measurements, the integrated photoluminescence was measured at varying excitation intensities and plotted as: 
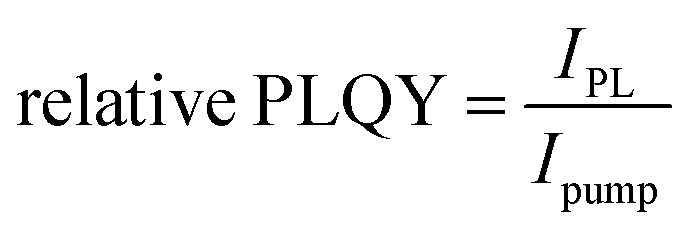
.

TRPL measurements were performed using a nitrogen cooled Hamamatsu Time Correlated Single Photon Counting (TCSPC) detector in a 50 ns measurement window, corresponding to a temporal resolution of 1 ns (FHM of the instrument response function). The decays were collected at a wavelength corresponding to the maximum of the PL spectrum. The sample was excited using a Chameleon oscillator (pulse duration 250 fs, 80 MH repetition rate) with central wavelength 800 nm; the repetition rate was reduced to 4 MHz through a pulse picker. The beam was focused on the sample with a 4 cm focal lens to a spot size of 15 μm radius. All measurements were performed in air on an encapsulated sample.

Absolute PLQY measurements were obtained from measurements performed in an integrating sphere (Labsphere) on encapsulated thin films deposited on non-conductive glass. Excitation was provided by a 405 nm c.w. diode laser (beam diameter ∼370 μm) and spectra acquired through an optical fiber coupled from the sphere to a spectrometer (Ocean Optics Maya Pro 2000) with an excitation power of 100 mW cm^−2^. PLQY values were calculated employing the method proposed by de Mello *et al.* (de Mello *et al.*, An improved experimental determination of external photoluminescence quantum efficiency, *Adv. Mater.*, 1997, **9**, 230–232). Error bars indicate 95% confidence intervals of 3 different measurements of the same sample.

Electrical conductivity (*σ*) measurements were obtained by depositing the perovskite film onto Au gold stripe contacts. *σ* was calculated as 
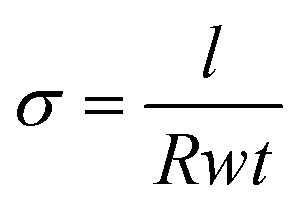
, where *l* is the length of the Au contacts (0.7 cm), *R* is the average resistance, *t* is the thickness of the perovskite film and *w* is the width between the 2 Au contacts (0.5 cm). The resistance *R* was measured by using a 2-point electrical probe. An Agilent B1500A Semiconductor Device Parameter Analyzer (SPA) was used to impose a voltage sweep from −1 V to 1 V between the two probes and the corresponding values of current were recorded.

### Computational details

We carry out hybrid DFT calculations at the PBE0 level^44,45^ of theory with non-local van der Waals interactions included through the rVV10 scheme,^46,47^ a method which has been tested in previous studies on metal halide perovskites.^14,47,48^ We employ the freely-available CP2K suite of codes.^49^ Goedecker–Teter–Hutter pseudopotentials are used to account for core–valence interactions^50^ while double-*ζ* polarized basis sets are adopted for the wave functions.^51^ We consider a cut-off of 600 Ry for the expansion of the electron density in plane waves. We employ the auxiliary density matrix method to speed up the calculation of exact exchange in hybrid functional calculations as implemented in CP2K with the cFIT auxiliary basis set.^52^ We note that spin–orbit coupling (SOC), while significantly contributing to the electronic properties of metal halide perovskite, affects only marginally those originating from the valence band edge. At variance with this, SOC impacts principally on the electronic properties associated with conduction band edge states.^53,54^ Therefore, we perform supplementary calculations with the QUANTUM ESPRESSO code employing full relativistic pseudopotentials on the structures achieved without SOC for systems in which extra electrons have been added, in line with previous studies.^55,56^

Calculations are performed on the archetypal tin perovskite, MASnI_3_. In particular, we consider the tetragonal phase which is stable at room temperature. Bulk calculations are performed on a 2 × 2 × 2 supercell consisting of 384 atoms and corresponding to the experimental density (has *a* = *b* = 17.5154 Å, *c* = 24.858 Å). Calculations of the (001) surface of tetragonal MASnI_3_ are performed on 5-layers slabs: (i) a 408-atoms slab terminated with lead diiodide (PbI_2_), (ii) a 552-atoms slabs terminated with methylammonium iodide (MAI) and (iii) a 552-atoms slab terminated with methylammonium fluoride (MAF). For all the considered slabs, the simulation cell has *a* = *b* = 17.5154 Å, *c* = 50 Å, including a vacuum layer up to 25 Å. The adequacy of the slab models employed in this work has been extensively tested in previous publications against slab thickness.^14,47,48^ The models are reported in Fig. S18 (ESI†). The calculated alignment of the valence band edge with respect to the vacuum level is reported in Table S4 (ESI†) for each surface termination.

Formation energies and charge transition levels of point defects are calculated with the grand-canonical formulation of defects in crystalline materials.^57^ In this theory, the formation energy of a defect *X* with charge *q*, *E*^*q*^_f_[*X*] is defined as a function of the electron chemical potential *μ*:S1
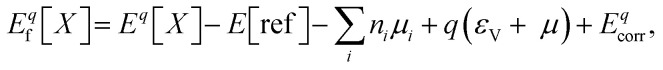
where, *E*^*q*^[*X*] is the total energy of the defect *X* in the charge state *q*, *E*[ref] the total energy of the pristine reference system (either bulk or slab), *μ*_*i*_ the chemical potential of the subtracted/added species *i*, *ε*_V_ the valence band edge of the pristine system, and *E*^*q*^_corr_ a correction term, here introduced to account for electrostatic finite-size effects of charged periodic supercells. For MASnI_3_, the chemical potentials of Sn and I, referenced with respect to metallic Sn and solid I_2_, have been defined in ref. 47. We here consider I-medium conditions, for which *μ*_Sn_ = −0.70 eV and *μ*_I_ = 0.51 eV. The electron chemical potential for which the formation energies of a defect *X* in the charge states *q* and *q*′ are equal 
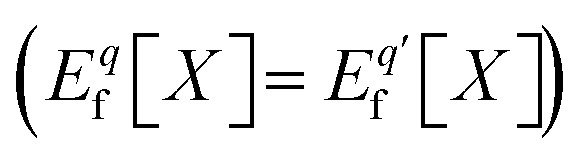
 is the charge transition level *μ*(*q*/*q*′) and read as follows:S2
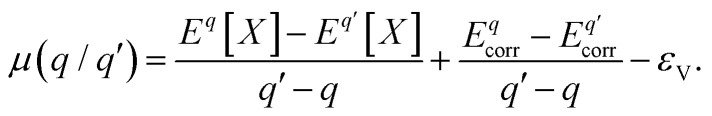
Electrostatic finite size corrections for slabs are here taken into account with the Freysoldt–Neugebauer–Van de Walle (FNV) scheme^58,59^ and, specifically, the method proposed by Komsa and Pasquarello^58^ is adopted for charged slabs. This scheme allows for a separation of the spurious interactions between the periodically repeated charges and the interactions between physical image charges occurring because of the variation in the dielectric constant across the surface. The energy correction is given as *E*^*q*^_corr_ = *E*_iso_ − *E*_per_ + *q*Δ*V*, where *E*_per_ is the electrostatic energy calculated for a model representing the employed supercell, *E*_iso_ the electrostatic energy obtained when uniformly scaling all the dimensions of the supercell, and Δ*V* the shift in the electrostatic potential between the model and the DFT calculation.

## Author contributions

AT performed simulations and experimental measurements, FA performed the theoretical calculations; SM, MDA and GF contributed to the design of the experiments; AJB contributed in the development of the simulation model; IP, synthetized the materials; AP and IP co-supervised the project. The first draft of the manuscript was prepared by AT, FA and IP with input from all the authors. All authors have contributed to the revision of the manuscript and have given approval to its final version.

## Conflicts of interest

There are no conflicts to declare.

## Supplementary Material

MH-009-D2MH00008C-s001
