# Supplementary material for: Effect of electronic doping and traps on carrier dynamics in tin halide perovskites
Source: Mater Horiz. 2022 Apr 1;9(6):1763–73. doi: 10.1039/d2mh00008c (PMC9390658; doi:10.1039/d2mh00008c)
Supplement: MH-009-D2MH00008C-s001 [file MH-009-D2MH00008C-s001.pdf]

Electronic Supplementary Material (ESI) for Materials Horizons.
This journal is © The Royal Society of Chemistry 2022

Supporting Information

Effect of Electronic Doping and Traps on Carrier Dynamics in Tin Halide Perovskites

Antonella Treglia[1,2], Francesco Ambrosio[1,3,4], Samuele Martani[1,2], Giulia Folpini[1], Alex J. Barker[1], Munirah D. Albaqam[6], Filippo De Angelis[3,5], Isabella Poli[1], Annamaria Petrozza[1,6*]*

1. Center for Nano Science and Technology @PoliMi, Istituto Italiano di Tecnologia, via G. Pascoli 70/3, 20133, Milano, Italy.

2. Physics Department, Politecnico di Milano, Piazza L. da Vinci, 32, 20133 Milano, Italy.

3.Computational Laboratory for Hybrid/Organic Photovoltaics (CLHYO), Istituto CNR di Scienze e Tecnologie Chimiche "Giulio Natta" (CNR- SCITEC), Perugia, Italy.

4. Department of Chemistry and Biology "A. Zambelli", University of Salerno, 84084 Fisciano, Salerno, Italy

5. Department of Chemistry, Biology and Biotechnology, University of Perugia, Perugia, Italy.

6. Chemistry Department, College of Science, King Saud University, Riyadh 11451, Saudi Arabia

e-mail: annamaria.petrozza@iit.it, isabella.poli@iit.it

S1. Kinetic model to simulate the evolution of the population of free and trapped carriers

S1.1. Simplification of the model

The recombination dynamics of charge carriers in a p-doped semiconductor can be described by the following rate equations:

$$\frac{dn_e}{dt} = G - k_2 n_e n_h - k_{1_e} n_e (N_{te} - n_{te}) - k_{dt_h} n_e n_{th} - k_{3h} n_e {n_h}^2 - k_{3e} n_h {n_e}^2 \quad \text{(Eq. S1)}$$

$$\frac{dn_h}{dt} = G - k_2 n_e n_h - k_{1_h} n_h (N_{th} - n_{th}) - k_{dt_e} n_h n_{te} - k_{3h} n_e {n_h}^2 - k_{3e} n_h {n_e}^2 \quad \text{(Eq. S2)}$$

$$\frac{dn_{te}}{dt} = k_{1e} n_e (N_{te} - n_{te}) - k_{dte} n_h n_{te} \quad \text{(Eq. S3)}$$

$$\frac{dn_{th}}{dt} = k_{1h} n_h (N_{th} - n_{th}) - k_{dth} n_e n_{th} \quad \text{(Eq. S4)}$$

Where n_e, n_h and n_{te}, n_{th} are respectively the density of free and trapped electrons and holes; G is the photogeneration rate. The total density of electron (N_{te}) and hole (N_{th}) traps is included taking into account the reduction of the total number of available traps as the population of trapped carriers increases with a cross-section k_{1e} and k_{1h} respectively for electrons and holes. The model also takes into account the possibility of an event of non-radiative recombination of a trapped carrier with its free counterpart. This can happen with cross-section k_{dte} in case of recombination of a free hole with a trapped electron and conversely with k_{dth}. Based on DFT calculations[1] deep electron traps appear to be the main contribution to the defect panel in Sn-based perovskites. For this reason, the model can be at a first stage simplified neglecting the contribution of deep hole traps. Auger recombination is included with a weight set by k_{3e} and k_{3h} respectively for eeh and hhe events.

When dealing with a pulsed laser, the generation term can be set to zero and the initial electron and hole populations are set as the density of absorbed photons right after a short pulse of excitation light. The time-dependent PL intensity for a single pulse excitation is therefore proportional to the total number of emitted photons given by equation (S5) and simulated in Figure S1.

$$n_{PL}(t) = k_2 n_e(t) n_h(t) \quad \text{(Eq. S5)}$$

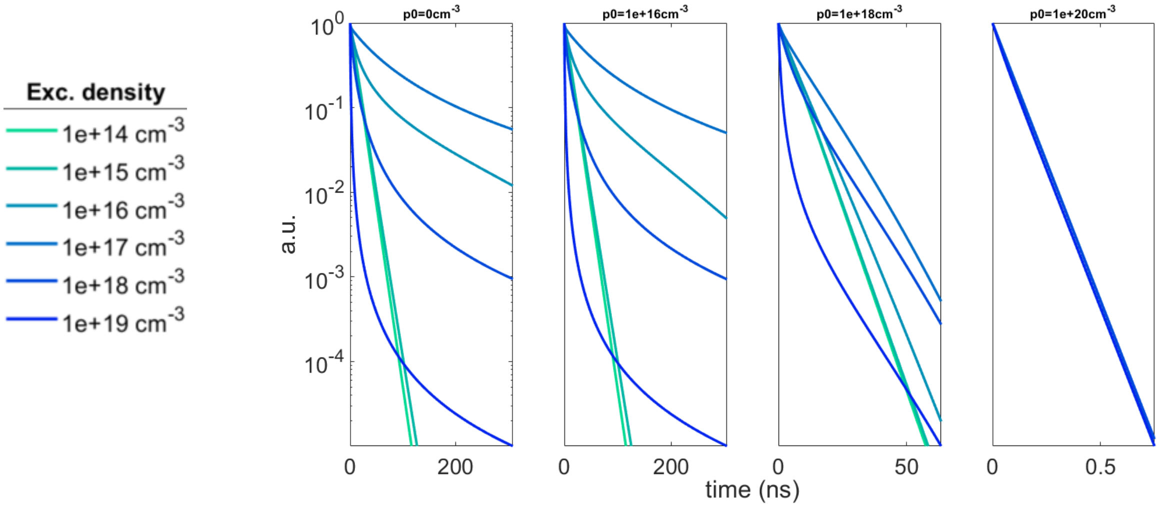

Figure S 1: Simulated TRPL decays for different excitation and doping densities. Main parameters from Table S 2.

Conversely, in the case of continuous wave (CW) excitation, the steady-state conditions can be simulated setting the initial populations to zero and defining the generation rate G as the absorbed photons over time according to the illumination conditions.

S1.2. Figures of merit

The carrier lifetime is extracted from the PL evolution with a delta function excitation and estimated as the time at which the PL intensity is decreased by a factor 1/e. The PLQY is the number of emitted photons normalized with respect to the excitation density $PLQY = \frac{n_{PL}}{n}$.

In this section we highlight the effects of the independent variation of the main parameters.

VARIABLE DOPING

Trapping rate electrons	$k_{1e} = 10^{-8} cm^3/s$
Radiative rate	$k_2 = 10^{-10} cm^3/s$
Recombination trapped electron with free hole	$k_{dte} = 10^{-11} cm^3/s$
Auger rate	$k_3 = 10^{-30} cm^6/s$
Total number of electron traps	$N_{te} = 10^{16} cm^{-3}$

Table S 1: Main parameters used for simulations in the *variable doping scenario* of the main text.

VARIABLE TRAP DENSITY

Trapping rate electrons	$k_{1e} = 10^{-8} cm^3/s$
Radiative rate	$k_2 = 10^{-10} cm^3/s$
Recombination trapped electron with free hole	$k_{dte} = 10^{-11} cm^3/s$
Auger rate	$k_3 = 10^{-30} cm^6/s$
Doping density	$p_0 = 10^{18} cm^{-3}$

Table S 2: Main parameters used for simulations in the *variable trap desity scenario* of the main text.

S1.3. Free parameter selection

a

RADIATIVE RATE

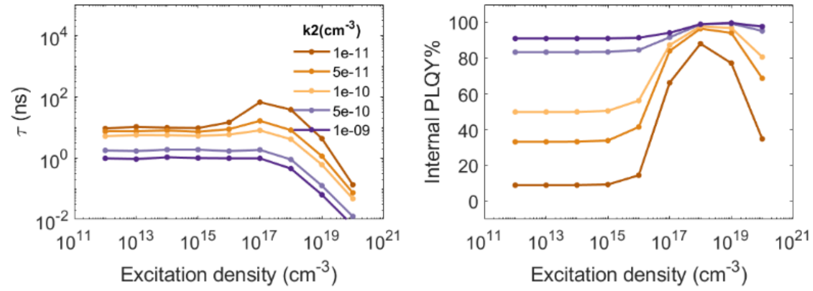

b

ELECTRON TRAPPING RATE

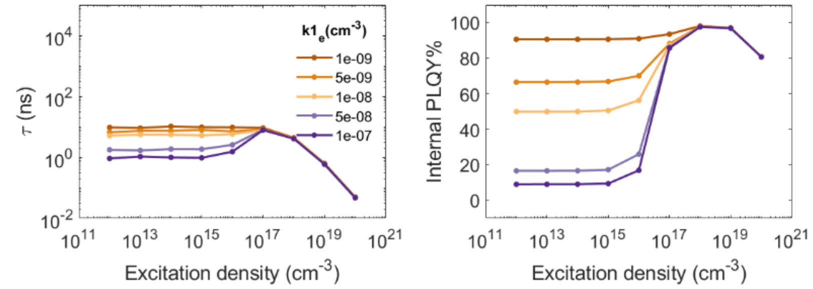

c

RATE OF RECOMBINATION OF A TRAPPED ELECTRON WITH A FREE HOLE

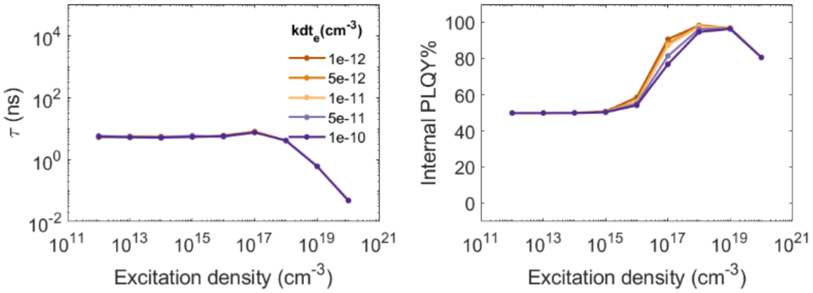

d

AUGER RATE

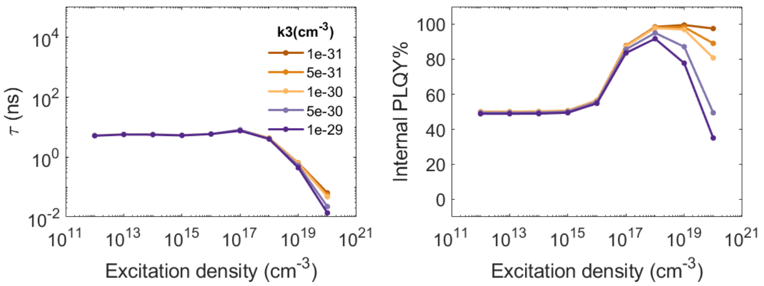

e

AUGER RATE

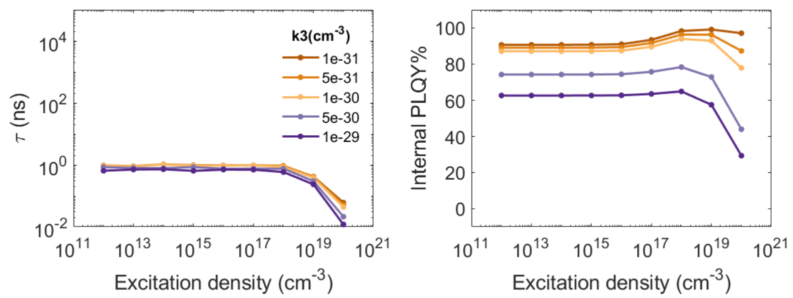

Figure S 2: Simulated lifetime and PLQY (500kHz) with parameters indicated in Table S 1 and doping density (a-d) p_0 = $10^{18} cm^{-3}$, (e) p_0 = $10^{19} cm^{-3}$. Each plot indicate the effect of the variation of one of the relevant parameters independently over two orders of magnitude: (a) radiative rate, (b) electron trapping rate, (c) rate of recombination of a trapped electron with a free hole, (d-e) Auger rate. From that we can deduce the impact a relative variation has in terms of carrier lifetime and PLQY either in the low (<10^{16}cm^{-3}) or high (>10^{16}cm^{-3}) excitation regime.

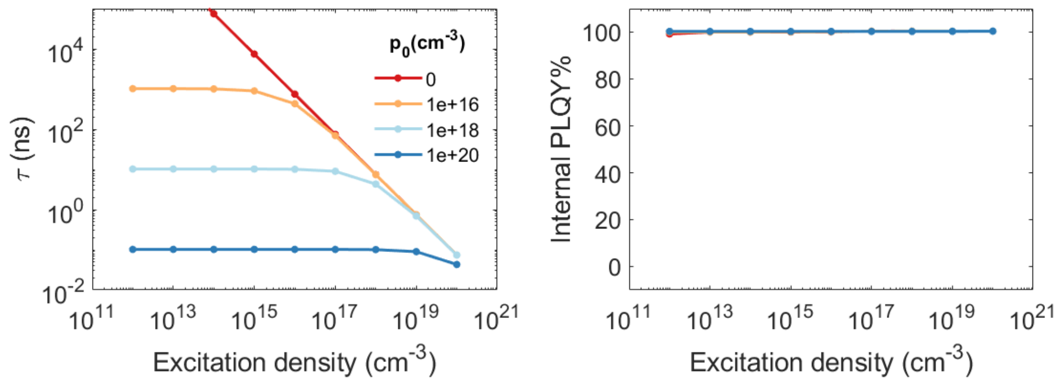

Trapping rate electrons	$-$
Radiative rate	$k_2 = 10^{-10} cm^3/s$
Recombination trapped electron with free hole	$-$
Auger rate	$-$
Total number of electron traps	$-$

Figure S 3: Simulated lifetime and PLQY (500kHz repetition rate) with only radiative recombination. Simulation parameters are indicated in the annexed table. PLQY is fixed at 100% independently from doping since there are no loss channels. Without doping the lifetime decreases with increasing excitation density due to band to band radiative recombination. With increasing doping the lifetime is decreased and it is flat up to an excitation density equal to the doping density. In this regime the pseudo-monomolecular recombination of electrons with dopant holes is dominant. Above the bimolecular recombination of photoexcited carries dominates.

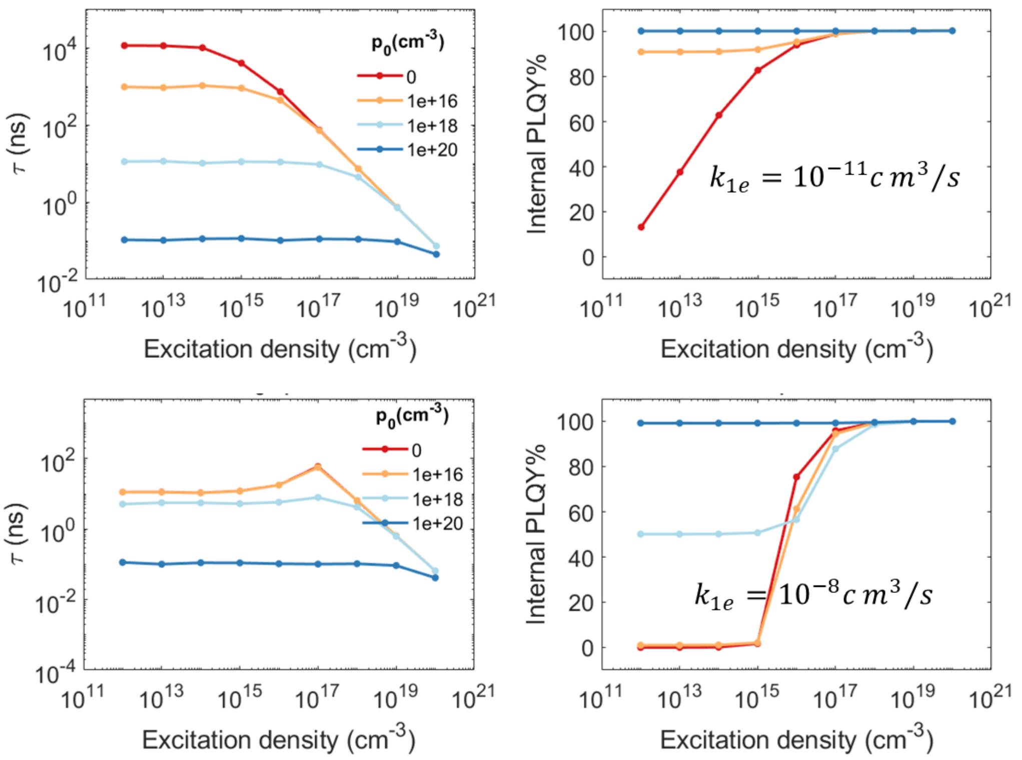

Trapping rate electrons	$variable$
Radiative rate	$k_2 = 10^{-10}cm^3/s$
Recombination trapped electron with free hole	$k_{dte} = 10^{-11}cm^3/s$
Auger rate	$-$
Total number of electron traps	$N_{te} = 10^{16}cm^{-3}$

Figure S 4: Simulated lifetime and PLQY (500kHz) with radiative recombination and trapping for electrons, Auger term is neglected. Simulation parameters are indicated in the annexred table. Top: slow electron trapping ($k_{1e} = 10^{-11}cm^3/s$), bottom: fast electron trapping ($k_{1e} = 10^{-8}cm^3/s$). If the trapping rate is lower than the radiative rate overall radiative dynamics are dominating. If the trapping rate is higher than the radiative rate in case of high doping (>1e18 cm^{-3}) the lifetime is not affected. For low doping (<1e18 cm^{-3}) the lifetime increases for increasing excitation density. Above the density of traps the lifetime follows the decay associated to radiative bimolecular band to band recombination. The PLQY is also reduced at low excitation density.

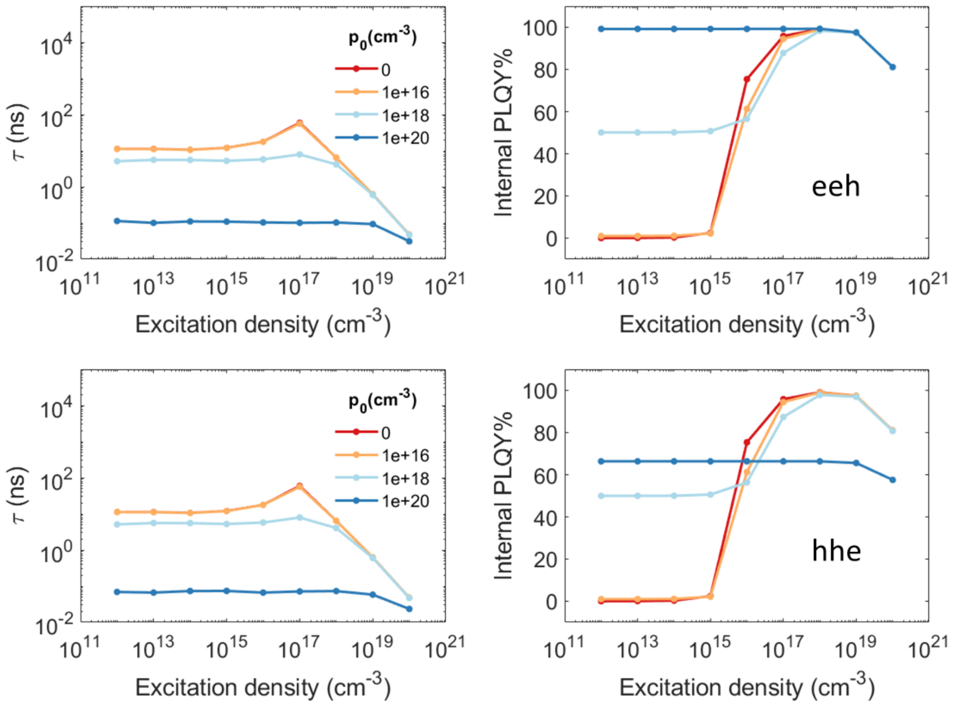

Trapping rate electrons	$k_{1e} = 10^{-8} cm^3/s$
Radiative rate	$k_2 = 10^{-10} cm^3/s$
Recombination trapped electron with free hole	$k_{dte} = 10^{-11} cm^3/s$
Auger rate	$k_3 = 10^{-30} cm^6/s$
Total number of electron traps	$N_{te} = 10^{16} cm^{-3}$

Figure S 5: Simulated lifetime and PLQY (500kHz) with radiative recombination and trapping for electrons, Auger is now included. Simulation parameters are indicated in the annexed table. Top: eeh processes $k_{4e} = 10^{-30} cm^6/s$, bottom: ehh processes $k_{4h} = 10^{-30} cm^6/s$. For doping densities above 10^{18}cm^{-3} Auger third order effects, that scale with the second power of hole density, limit the radiative efficiency, causing a reduction in PLQY and almost flat behavior over different orders of magnitude in excitation density.

S2. Spectroscopic measurements

a b c

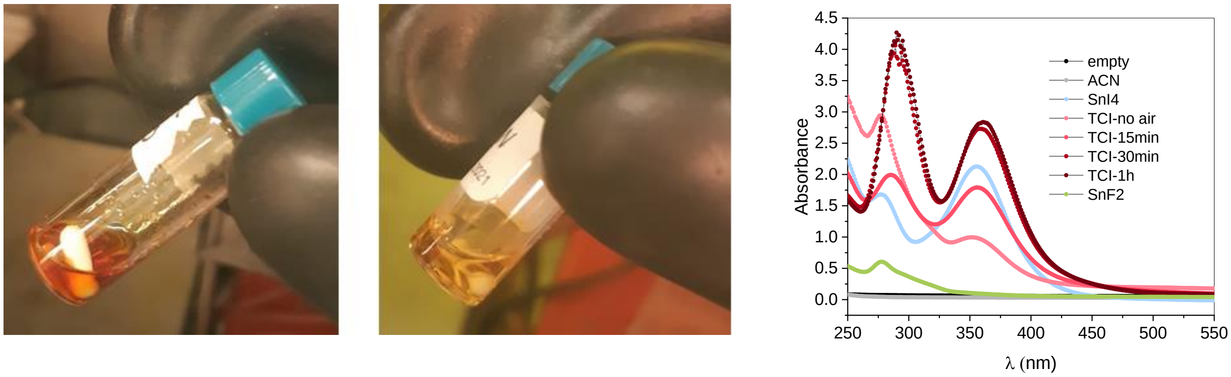

Figure S6: Pictures of solutions of $FACsSnI_3$ (a) with and (b) without SnI_4. The red component is an indication of the content of Sn^{4+}, (c) absorbance of solution of SnI_4, SnI_2 oxidised in air and SnI_2 oxidised with subsequent addition of SnF_2. All solutions were 0.14 molar in acetronitrile and measured in a sealed quartz cuvette.

a

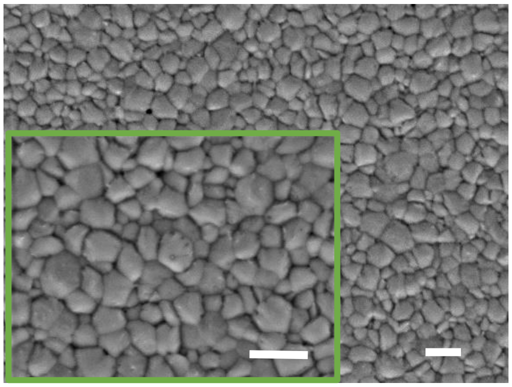

b

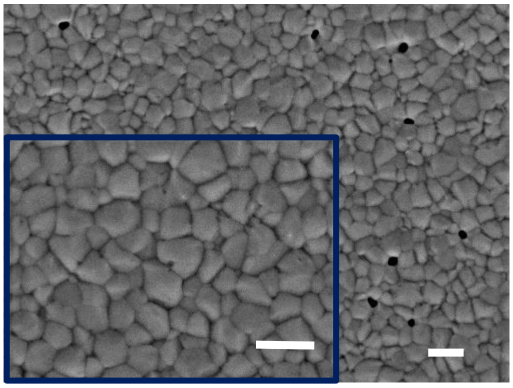

c

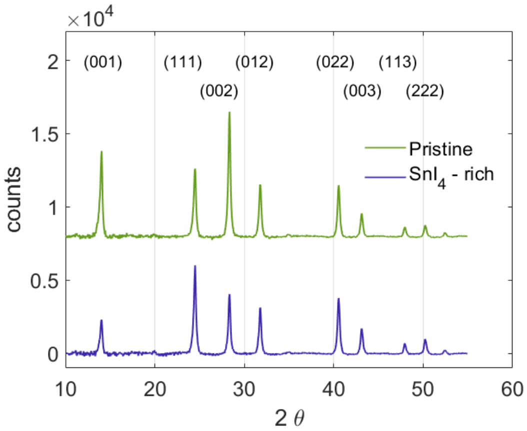

Figure S7**:** Top view SEM images of (a) pristine (scale bar 1µm) and (b) SnI4-right (scale bar 1µm) thin film perovskites, respectively. (c) XRD patterns of pristine and SnI_4-rich thin film perovskites. The measurement was carried out in Nitrogen inert atmosphere to prevent degradation due to air exposure. both samples are highly crystalline and present well-defined and sharp peaks at 14°, 24.5°, 28.5°, 32°, 40.5°, 43°, 48°and 50°, which correspond to the (001), (111), (002), (012), (022), (003), (113) and (222) phases, respectively, typical of a pseudocubic crystal structure, as previously reported.[2,3] The absence of a shift of the peaks suggests that the lattice constant is not modified by the presence of SnI_4. The additive affects the relative intensity of the peaks resulting in a decrease for (001), (002) and (012) in favor of a preferential growth for (111) plane. Neither broadening of diffraction peaks nor the presence of impurity phases are observed, suggesting that Sn^{4+} does not induce any clear lattice distortion.

a b

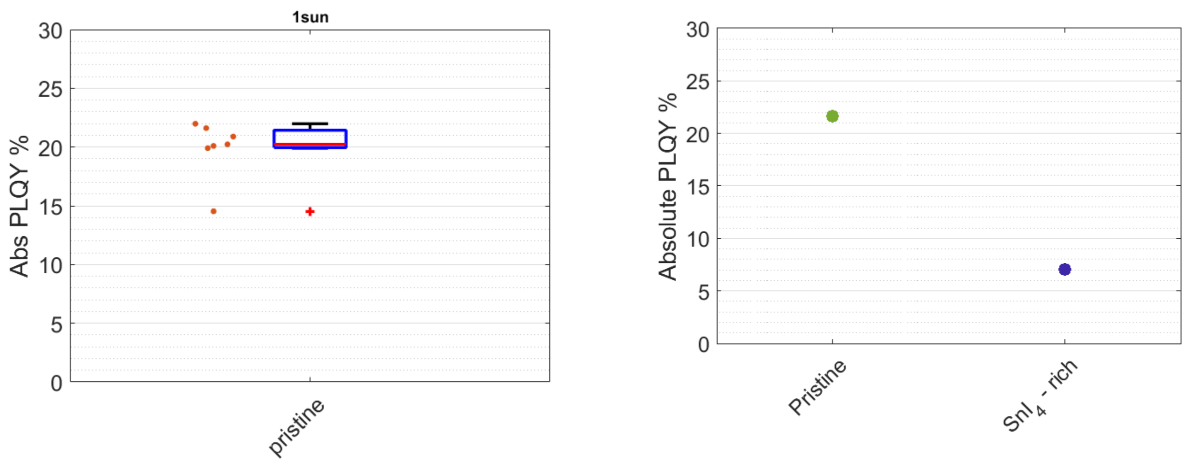

Figure S 8: a)Average absolute PLQY over 7 pristine samples all fabricated in different batches, b) absolute PLQY of two samples fabricated in the same conditions from a pristine and SnI_4-rich solution.

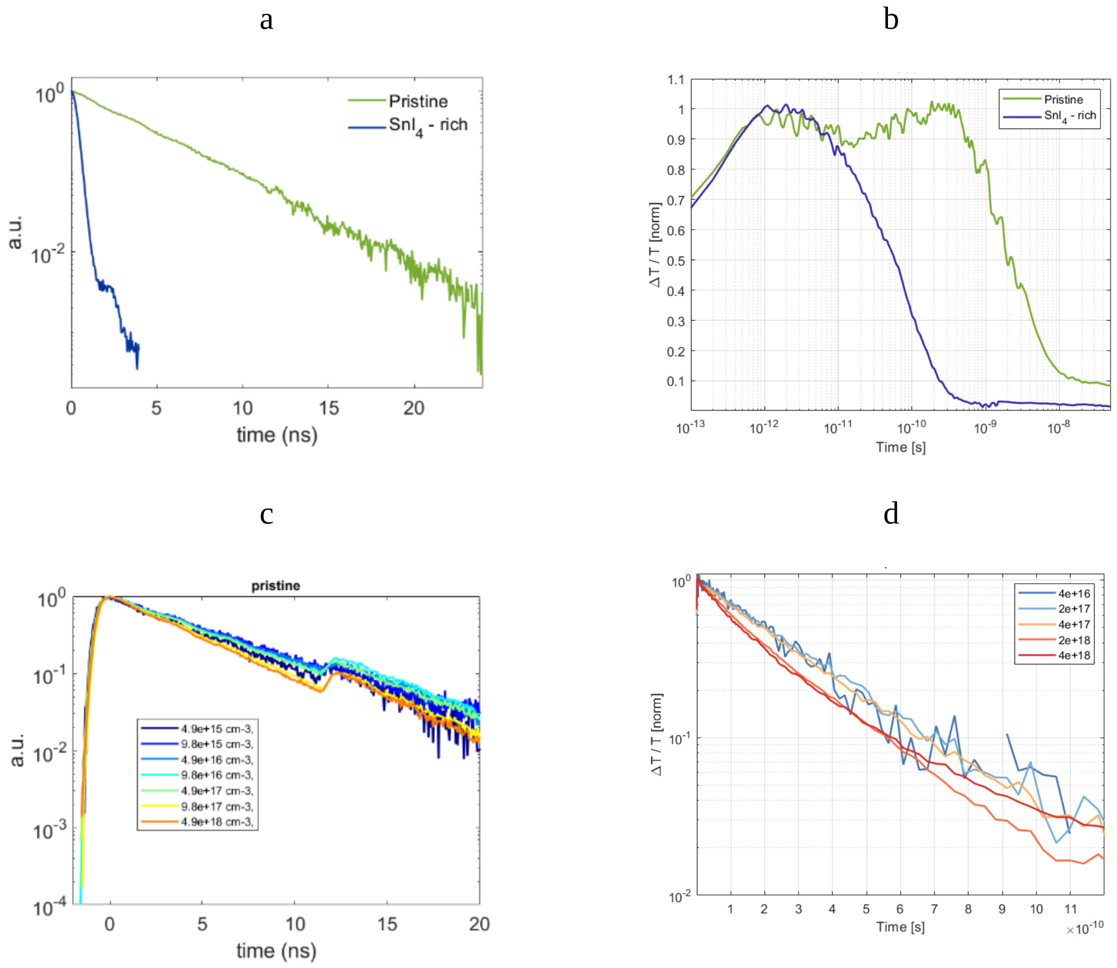

Figure S9: a) TRPL decay for the pristine and SnI_4-rich samples. Measured with excitation wavelength of 800 nm and density of about 5e17cm^{-3} the decay of the SnI_4-rich sample is not resolved b) Transient Absorption Spectroscopy measurements describing the recovery of the main photobleach (840-880nm) in the short delays range (<5ns) for the pristine and SnI_4-rich samples, c) fluence dependent TRPL decay of the pristine sample, d) Transient Absorption Spectroscopy measurements describing the recovery of the main photobleach (840-880nm) in the short delays range (<5ns) for the SnI_4-rich sample as function of pump excitation density.

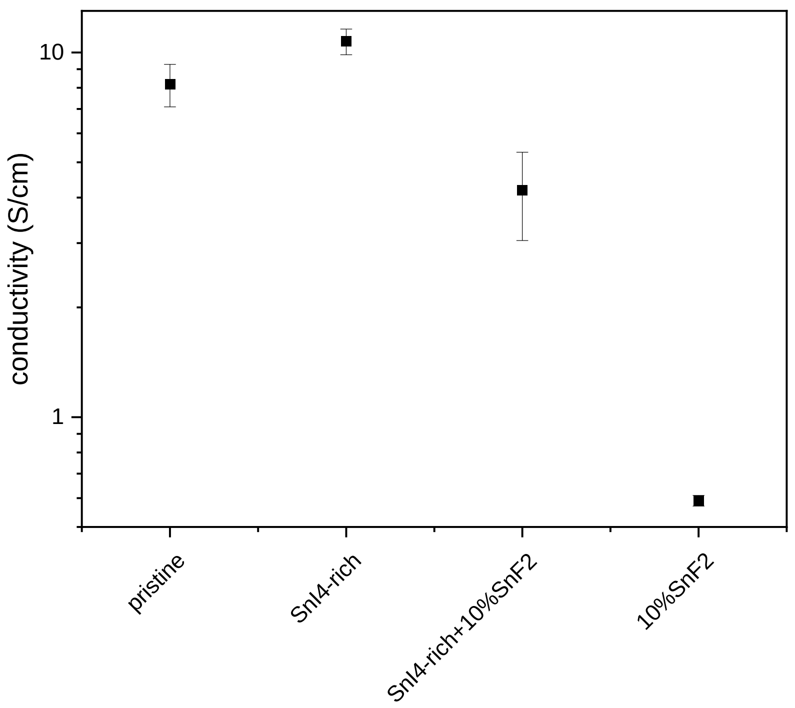

Figure S 10: Conducibility of pristine, SnI_4-rich, SnI_4-rich + 10% SnF_2 and 10% SnF_2 samples measured in nitrogen.

a b

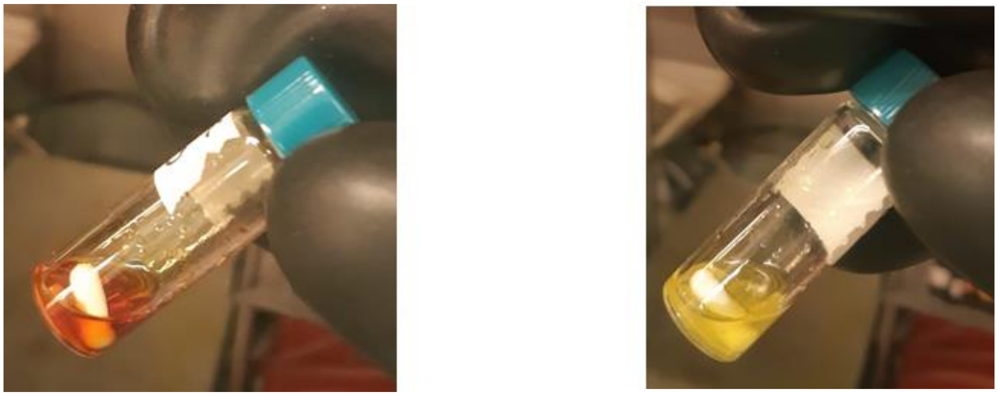

Figure S11: Pictures of solutions of $FACsSnI_3$ (a) with SnI_4 (b) 10% SnF_2. The red component is an indication of the content of Sn^{4+}.

a

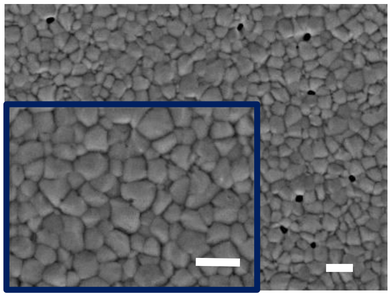

b

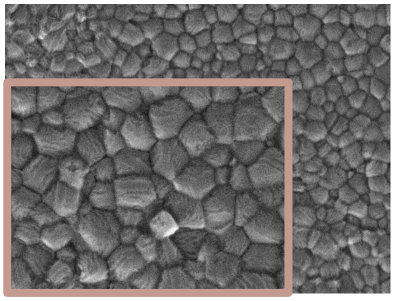

c

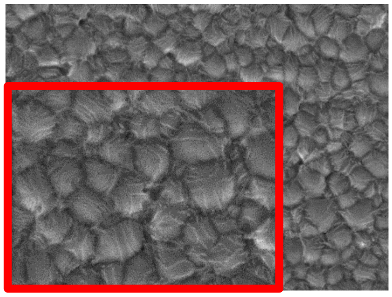

d

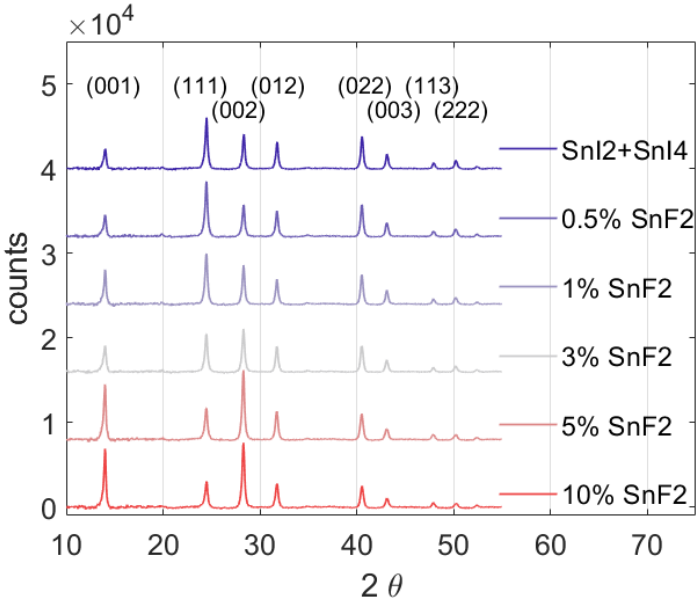

Figure S 12: a-b-c Top view SEM images of SnI_4-rich (left panel), with 5% SnF_2 (central panel) and 10% SnF_2 (right panel). The scale bars are the same for all images and both correspond to 1µm. d. XRD patterns of the reference SnI_4-rich film and films with gradual increase of SnF_2. SnF_2 affects the relative intensity of the XRD peaks resulting in a decrease for (111), (022) and (003) in favor of a preferential growth for (001) and (002) planes that are those perpendicular to the plane of the substrate.

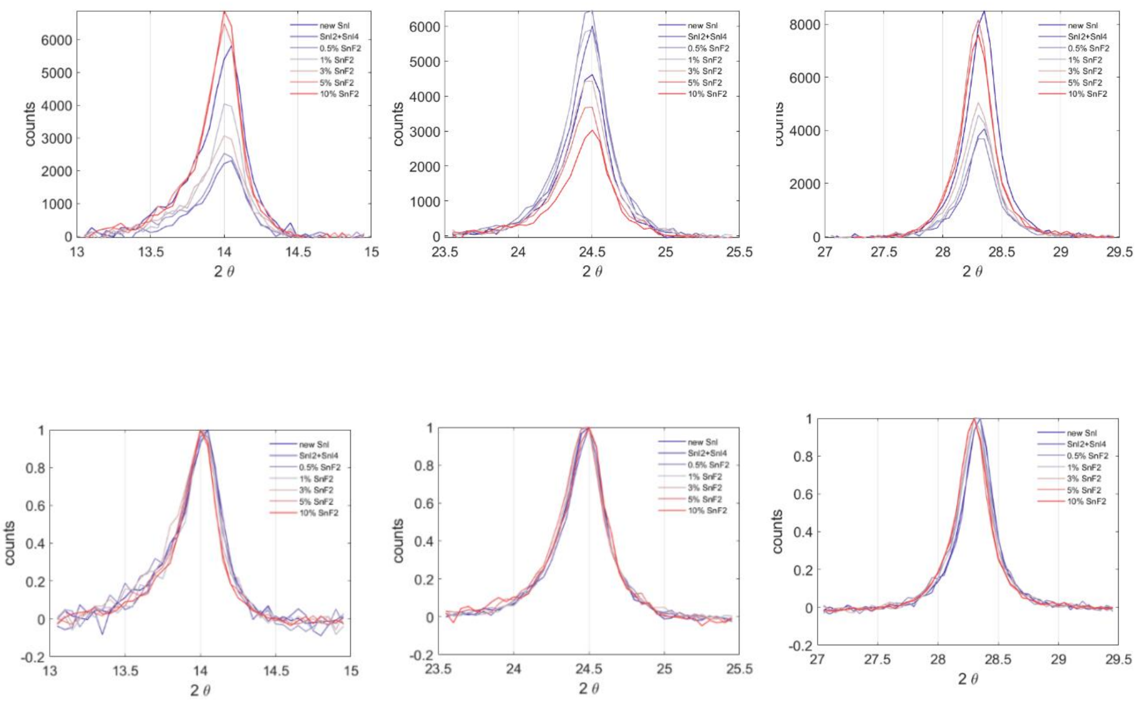

Figure S 13: Detail of the main peaks of the XRD patterns of the SnI_4-rich film and films with gradual increase of SnF_2. Minimum shift of the (001) and (002) peaks can be associated with a reduction in strain arising from Sn vacancies. Also is this case no tetragonal distortion is observed.

a b c

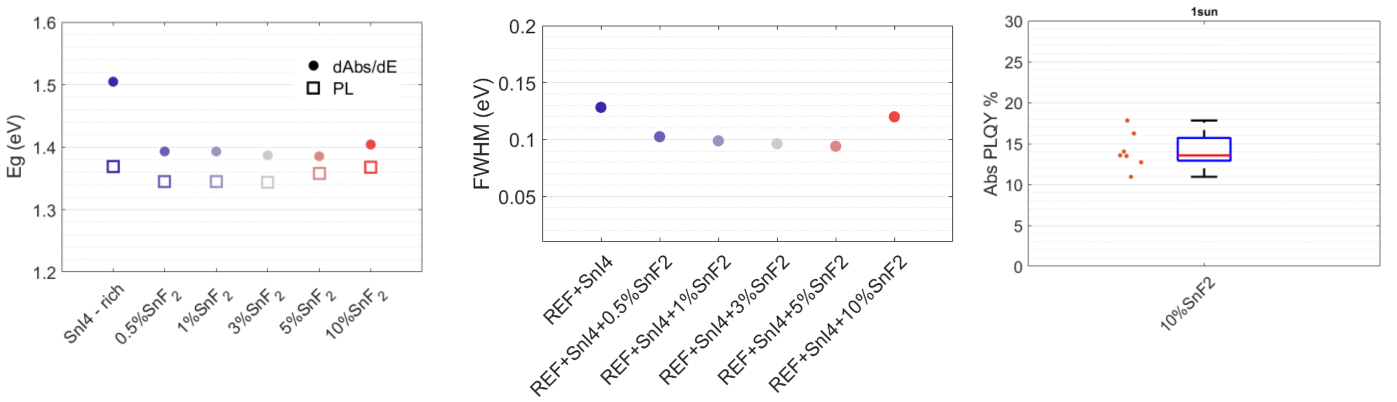

Figure S14: (a) dAbs/dE indicated with a dot and the maximum in the PL emission indicated with an empty square give also a visual indication of the Stoke shift. (b) Full width at half maximum of the PL peak the SnI2+SnI4 and with gradual increase of SnF_2, (c) Average PLQY over 7 samples all fabricated in different batches.

a b

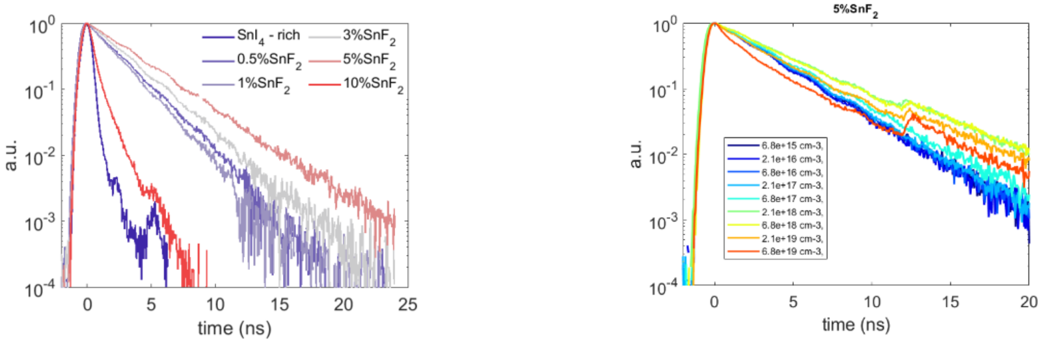

Figure S 15: a) TRPL decays of the samples SnI_4-rich and with gradual increase of SnF_2 content with excitation density of about 5e17cm^{-3}, b) fluence dependence of the sample with 5%SnF_2 added, at higher excitation densities is visible the emergence of a biexponential decay.

a b

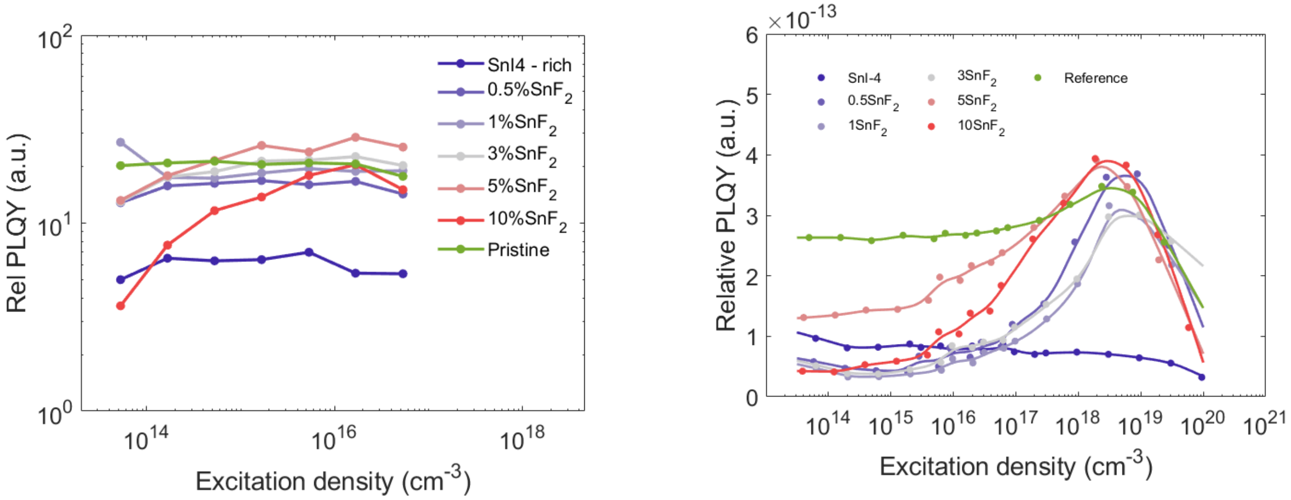

Figure S16**:** Relative PLQY not normalized, taken with increasing excitation intensities for all samples: (a) with 450 nm CW excitation (b) with 515nm pulsed excitation and 500kHz repetition rate.

REFERENCE

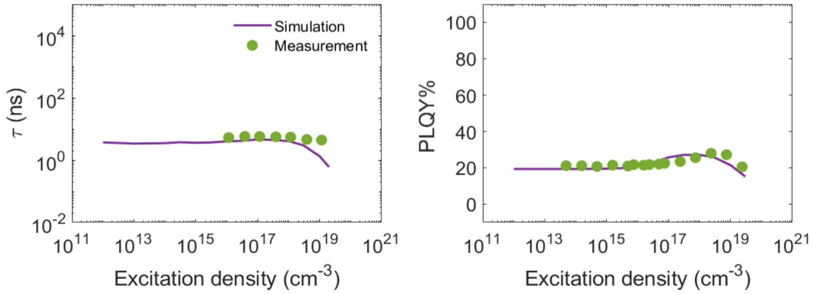

SnI_4-Rich

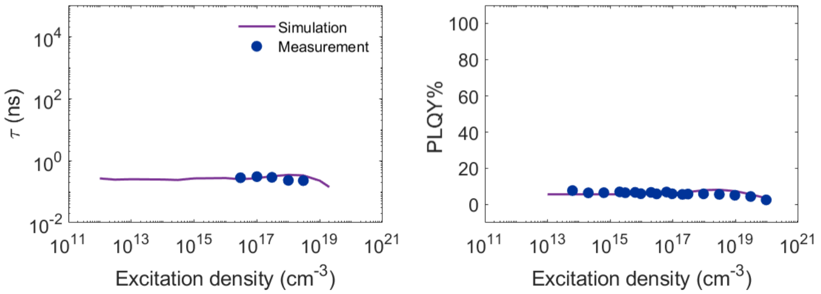

0.5%SnF_2

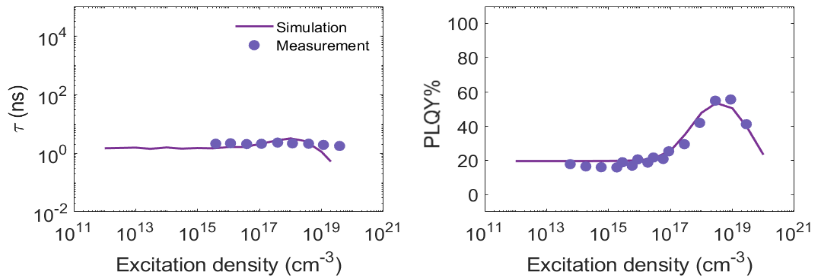

5%SnF_2

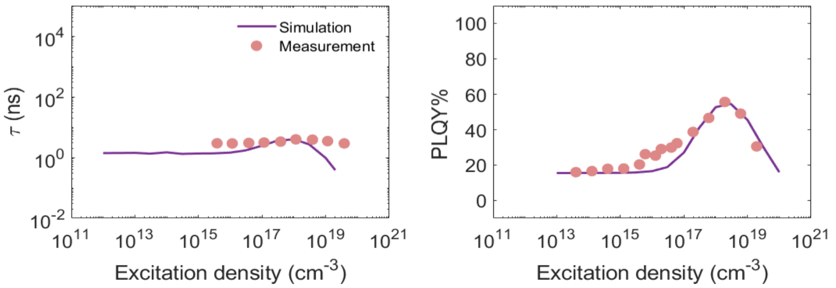

10%SnF_2

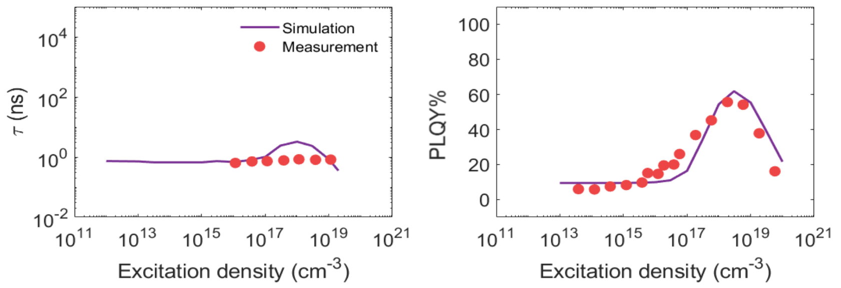

Figure S17: Dots indicate experimental lifetime and external PLQY measured with pulsed excitation (repetition rate 500kHz) and reported in Figure 2b-c and 3c-d of the main text. Continuous lines indicate simulated curves as a result of the simultaneous fitting of lifetime and PLQY, the parameters for each simulated curve are indicated in Table S3.

	**Reference**	**SnI_4**	**0.5%SnF_2**	**5%SnF_2**	**10%SnF_2**
**Trapping rate electrons k_{1e} $[cm^3/s]$**	$2 \cdot 10^{-8}$	$2 \cdot 10^{-8}$	$1 \cdot 10^{-8}$	$1 \cdot 10^{-8}$	$2 \cdot 10^{-8}$
**Radiative rate k_2 $[cm^3/s]$**	$0.6 \cdot 10^{-11}$	$0.6 \cdot 10^{-11}$	$2 \cdot 10^{-11}$	$2 \cdot 10^{-11}$	$3 \cdot 10^{-11}$
**Recombination trapped e$^-$ with free h $k_{dte}[cm^3/s]$**	$1 \cdot 10^{-11}$	$1 \cdot 10^{-11}$	$7 \cdot 10^{-11}$	$7 \cdot 10^{-11}$	$6 \cdot 10^{-11}$
**Auger rate k_3 $[cm^6/s]$**	$3 \cdot 10^{-30}$	$3 \cdot 10^{-30}$	$3 \cdot 10^{-30}$	$3 \cdot 10^{-30}$	$3 \cdot 10^{-30}$
**Total number of e$^-$ traps N_{te} $[cm^{-3}]$**	$5 \cdot 10^{15}$	$8 \cdot 10^{16}$	$5 \cdot 10^{16}$	$3 \cdot 10^{16}$	$7 \cdot 10^{16}$
**Doping p_0 $[cm^{-3}]$**	10^{19}	$4 \cdot 10^{19}$	$7 \cdot 10^{18}$	$6 \cdot 10^{18}$	$5 \cdot 10^{18}$

Table S3: Simulation parameters obtained from the simultaneous fitting of carrier lifetime and external PLQY shown in Figure S17.

S3. Surface models and band alignment

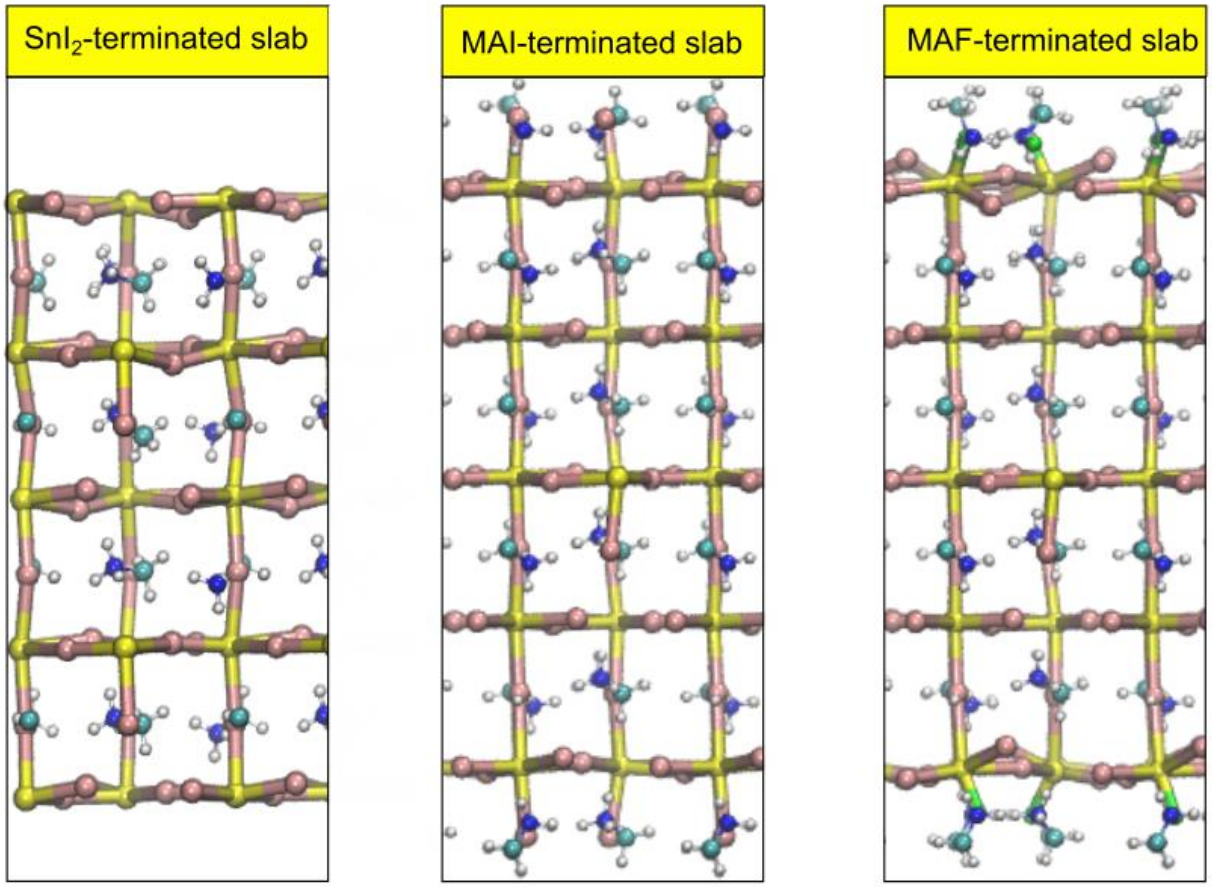

Figure S 18: Stick&ball representation (side-view) of the different slabs considered in this study. Sn atoms are given in yellow, I in pink, F in green, C in cyan, N in blue and H in white.

Surface termination	VBM Vs vacuum level (eV)
SnI_2	6.09
MAI	5.15
MAF	4.60

Table S 4: Valence band maximum (VBM) of MASnI3 aligned with respect to the vacuum level for each considered termination of the (001) surface of tetragonal MASnI3.

S3.2 Formation energies of point defects for the considered surfaces

a

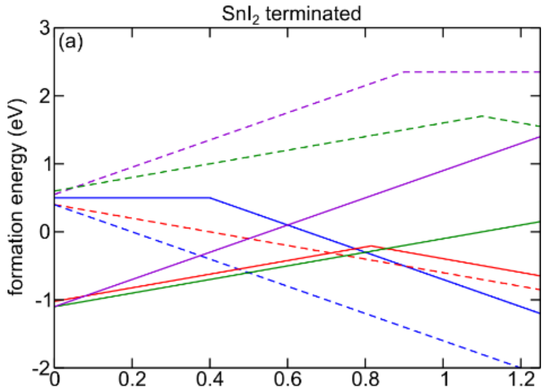

b

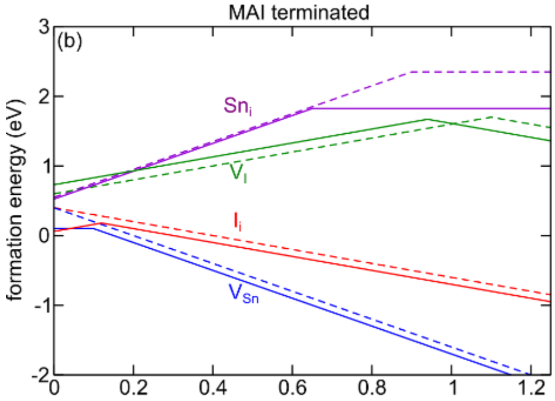

c

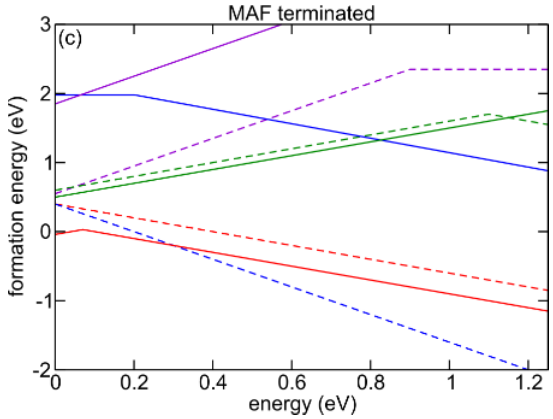

Figure S 19: Formation energies of point defects as a function of the electron chemical potential μ on the (a) SnI_2 (b) MAI and (c) MAF (001) terminated surfaces of tetragonal $MASnI_3$. Dashed lines for the respective values calculated for the bulk material. We consider iodine medium conditions as defined in Ref.[4]

S3.3 Atomistic models of selected point defects for different surface terminations

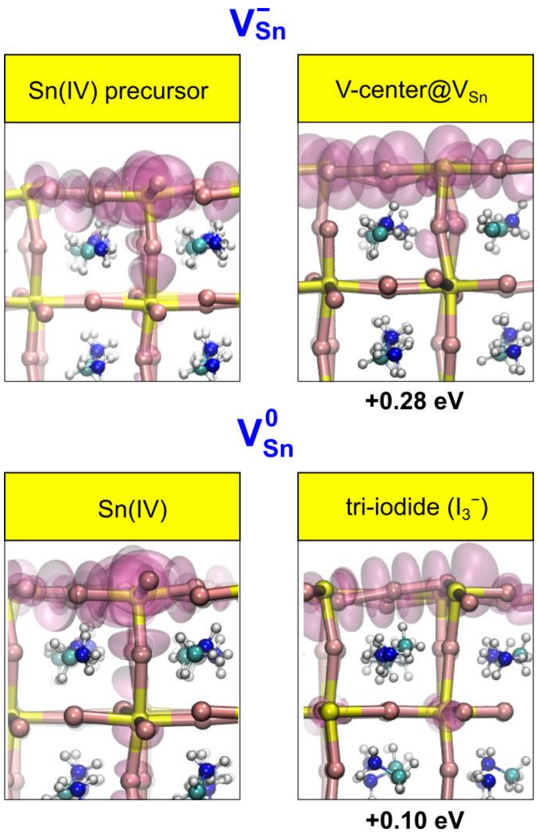

Figure S 20: Stick&ball representation (side-view) of different structural configurations for a surface Sn vacancy with one extra hole (V_{Sn}^{-}, top panels) and two extra holes (V_{Sn}^{0}, bottom panels). Left (right) panels for configurations entailing tin (iodide) oxidation. For each configuration, the isodensity of the lowest unoccupied molecular orbital is given in violet and the energy difference is reported, as referred to the most stable configuration.

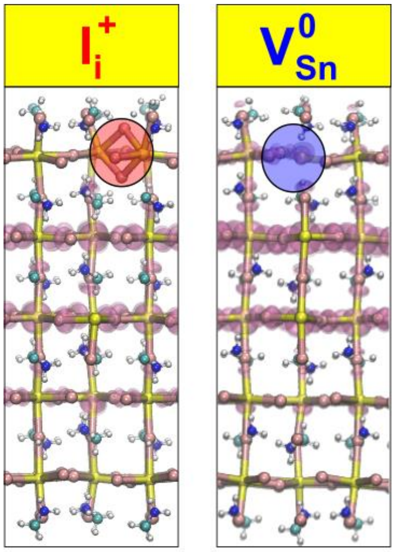

Figure S 21: Stick&ball representation of the surface I interstitial (left panel) and Sn vacancy (right panel) with two extra holes on the MAI terminated (001) surface of $MASnI_3$. For each configuration, the isodensity of the lowest unoccupied molecular orbital is given in violet and the position of the defect is highlighted with a shaded circle.

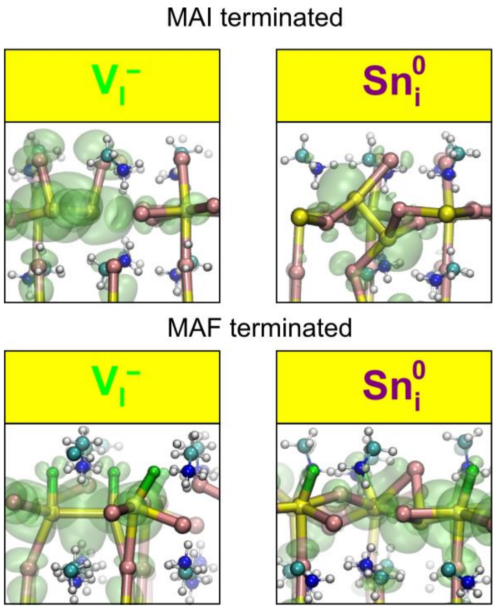

Figure S 22: Stick&ball representation of the surface I vacancy and Sn interstitial with two extra electrons, V_I^- and Sn_i^0, respectively on the MAI terminated (top) and MAF terminated (bottom) (001) surfaces of $MASnI_3$. For each configuration, the isodensity of the highest occupied molecular orbital is given in green.

MAI terminated slab		
Defect	E_b (eV)	$d_{\text{Sn-Sn}}$ (Å)
Sn_i^0	0.25	3.03
V_I^-	0.14	3.25
**MAF terminated slab**		
Defect	E_b (eV)	$d_{\text{Sn-Sn}}$ (Å)
Sn_i^0	0.010	3.43
V_I^-	−0.21	3.46

Table S 5: Calculated binding energies (eV) of the Sn-Sn dimer formed upon trapping of two electrons along with bond length (Å) for different defective models of the MAI and MAF terminated surfaces of tetragonal $MASnI_3$.

S4. References

1. Meggiolaro, D., Ricciarelli, D., Alasmari, A. A., Alasmary, F. A. S. & De Angelis, F. Tin versus Lead Redox Chemistry Modulates Charge Trapping and Self-Doping in Tin/Lead Iodide Perovskites. *J. Phys. Chem. Lett.* **11**, 3546–3556 (2020).

2. Savill, K. J. *et al.* Impact of Tin Fluoride Additive on the Properties of Mixed Tin-Lead Iodide Perovskite Semiconductors. *Adv. Funct. Mater.* **2005594**, 1–13 (2020).

3. Dang, Y. *et al.* Formation of Hybrid Perovskite Tin Iodide Single Crystals by Top-Seeded Solution Growth. *Angew. Chemie Int. Ed.* **55**, 3447–3450 (2016).

4. Meggiolaro, D., Ricciarelli, D., Alasmari, A. A., Alasmary, F. A. S. & De Angelis, F. Tin versus Lead Redox Chemistry Modulates Charge Trapping and Self-Doping in Tin/Lead Iodide Perovskites. *J. Phys. Chem. Lett.* **11**, 3546–3556 (2020).
